# Fibroblast Growth Factor Binding Protein 3 (FGFBP3) impacts carbohydrate and lipid metabolism

**DOI:** 10.1038/s41598-018-34238-5

**Published:** 2018-10-29

**Authors:** Elena Tassi, Khalid A. Garman, Marcel O. Schmidt, Xiaoting Ma, Khaled W. Kabbara, Aykut Uren, York Tomita, Regina Goetz, Moosa Mohammadi, Christopher S. Wilcox, Anna T. Riegel, Mattias Carlstrom, Anton Wellstein

**Affiliations:** 1 Lombardi Comprehensive Cancer Center, Department of Oncology, Georgetown University, School of Medicine, Washington, DC 20007 USA; 2 Department of Biochemistry and Molecular Pharmacology, New York University School of Medicine, New York, NY 10016 USA; 3 Division of Nephrology and Hypertension, Kidney, and Vascular Research Center, Georgetown University, School of Medicine, Washington, DC 20007 USA; 4 Department of Physiology & Pharmacology, Karolinska Institutet S-17177, Stockholm, Sweden

## Abstract

Secreted FGF binding proteins (FGFBP) mobilize locally-acting paracrine FGFs from their extracellular storage. Here, we report that FGFBP3 (BP3) modulates fat and glucose metabolism in mouse models of metabolic syndrome. BP3 knockout mice exhibited altered lipid metabolism pathways with reduced hepatic and serum triglycerides. In obese mice the expression of exogenous BP3 reduced hyperglycemia, hepatosteatosis and weight gain, blunted de novo lipogenesis in liver and adipose tissues, increased circulating adiponectin and decreased NEFA. The BP3 protein interacts with endocrine FGFs through its C-terminus and thus enhances their signaling. We propose that BP3 may constitute a new therapeutic to reverse the pathology associated with metabolic syndrome that includes nonalcoholic fatty liver disease and type 2 diabetes mellitus.

## Introduction

Fibroblast growth factor binding proteins (BP1, 2 and 3) are secreted chaperones known to bind and mobilize paracrine FGFs from their heparan sulfate (HS) storage in the extracellular matrix^1–4^. BP1, the best characterized member of this family, is known to interact with paracrine FGFs, such as FGF1, 2, 7, 10, and 22^2,3^ and to compensate for the lack of HS to enhance cellular FGF receptor signaling^5^. BP1 can modulate FGF functions during development, tumorigenesis, tissue homeostasis and tissue repair. BP1 knock-down induced early chick embryo lethality, whereas transient overexpression of BP1 caused aberrant vascular leakage^6,7^. BP1 expression is elevated in a range of cancers and it is rate-limiting for angiogenesis-dependent cancer growth and metastasis^8–12^. In addition, BP1 is an early response gene that is upregulated in wounded skin^13^, in regenerating renal tubular epithelial cells after hemolytic uremia syndrome^5^, in the aorta during early atherogenesis^14^, in nervous fibers after spinal cord injury^15^ as well as in myofibers during reinnervation in a model of amyotrophic lateral sclerosis (ALS)^16^. Gain-of-function studies in transgenic mice revealed that BP1 enhances neoangiogenesis in subcutaneous matrigel plugs, in ischemic muscle injury, as well as during skin wound healing^17^ and increases blood pressure^18^. Complementary to this, in loss-of-function studies, BP1^−/−^ mice displayed reduced angiogenesis, delayed wound healing and a blunted carcinogen-induced skin papillomatosis^19^.

Mechanisms of action and some of the biological effects of BP2, a gene lost in rodents, and of BP3 overlap with BP1 in that they function as chaperones for heparin-binding, paracrine FGFs and enhance FGF signaling^4,6^. However, potential interactions of the BPs with members of the non-heparin-binding, endocrine FGF19 family are unknown. These endocrine FGFs, namely FGF19, FGF21 and FGF23, are released into the circulation and control metabolic homeostasis of glucose, lipids and phosphate^20–22^. Here we describe the crosstalk of endocrine FGFs with BP3. BP3 knockout mice exhibited an altered lipid metabolism, with reduced serum and liver triglycerides. On the other hand, exogenous expression of BP3 in obese mice reduced body weight, hyperglycemia and normalized hepatic steatosis due to the suppression of lipogenic gene expression in the liver and white adipose tissue (WAT). Finally, we show that BP3 interacts with endocrine FGFs and modulates FGF19 and FGF21 signaling *in vitro*. Our data suggest a surprising contribution of BP3 to metabolic control that could provide an innovative treatment of diseases associated with the metabolic syndrome including nonalcoholic fatty liver disease (NAFLD) and type 2 diabetes mellitus^23,24^.

## Results

### BP3^−/−^ mice show reduced serum and liver triglycerides

To examine the effect of BP3 *in vivo*, we generated BP3^−/−^ mice from heterozygous founders obtained from the Knock-Out Mouse Project (KOMP) (Supplemental Fig. 1A–C). Offspring were viable, born at normal Mendelian ratios, with normal life spans and no gross abnormalities. An initial analysis performed by KOMP revealed that BP3^−/−^ mice exhibit a worsened GTT and lower circulating triglycerides (TG) (Supplemental Fig. 1D), suggestive of altered metabolic functions.

**Figure 1:**
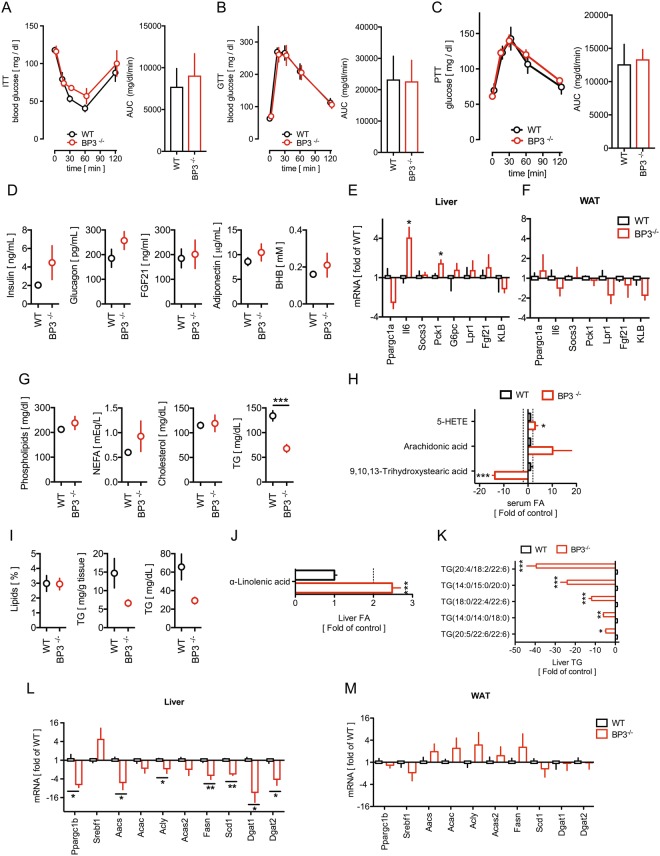
BP3^−/−^ mice show reduced serum and liver triglycerides. (**A**–**C**) Insulin tolerance test (ITT) (**A**); Glucose tolerance test (**B**) and Pyruvate tolerance test (**C**) and the respective Area under the curve (AUC) of WT and BP3^−/−^ mice. Data were analyzed with two-way ANOVA followed by Bonferroni post tests; mean ± SEM; *P < 0.05; **P < 0.01 vs. WT control. (**D**) Insulin, glucagon, Fgf21, adiponectin and BHB serum concentrations of WT and BP3^−/−^ mice. (**E**,**F**) Expression of genes involved in glucose homeostasis in livers (**E**) and WAT (**F**) from WT and BP3^−/−^ mice determined by qRT-PCR. (**G**) Phospholipids, non-esterified fatty acids (NEFA), cholesterol, and overall triglycerides (TG) serum concentrations of WT and BP3^−/−^ mice. (**H**) Metabolomics analysis of serum fatty acids. (**I**) Hepatic lipids and TG levels in WT and BP3^−/−^ mice. (**J**,**K**) Metabolomics analysis of linolenic acid (**J**) and TG isomers (**K**) in WT and BP3^−/−^ mice. (**L**,**M**) Expression of genes involved in lipogenesis in livers and WAT from WT and BP3^−/−^ mice. Data are expressed as fold of WT control (**E**,**F**,**L**,**M**). Data are expressed as mean ± SEM; n = 6–10, *P < 0.05; **P < 0.01; ***P < 0.0001 vs WT control.

To evaluate the metabolic state of the animals, we subjected BP3^−/−^ and wild type (WT) littermates to insulin, glucose and pyruvate tolerance tests but found no significant differences between the WT and BP3^−/−^ groups (Fig. 1A–C). Interestingly, the average serum insulin levels were not different between BP3^−/−^ mice and their WT littermates, but we observed a significantly higher degree of variance (F-test) in the BP3^−/−^ group with 50% of the mice exhibiting high insulin levels. Circulating levels of FGF21, a potent insulin sensitizer and metabolism regulator, are elevated in mouse models of obesity although obese mice still respond to high doses of FGF21 by ameliorating glucose and lipid parameters (reviewed in^20^). In our study FGF21 levels were low and undistinguishable between WT and BP3^−/−^ mice and the levels of glucagon, adiponectin or beta-hydroxybutyrate (BHB) were unchanged between the two groups (Fig. 1D). The liver and WAT are major regulators not only of energy storage in the body, but also of the control of circulating fatty acids and various lipids, as well as of glucose homeostasis^25^ and we thus evaluated gene expression of enzymes that control glucose metabolism in livers and white adipose tissues (WAT) of BP3^−/−^ and WT mice. Loss of BP3 resulted in a significant mRNA upregulation of hepatic phosphoenolpyruvate carboxykinase 1 (*Pck1*), a key regulator of gluconeogenesis, and of interleukin 6 (*Il6*) that has been linked to insulin resistance^26,27^ (Fig. 1E). Hepatic peroxisome proliferator-activated receptor-γ coactivator-1α (*Ppargc1a*), suppressor of cytokine signaling 3 (*Socs3*), glucose-6-phospatase (*G6pc*), Leptin receptor 1 (*Lpr1*), *Fgf21* and β-Klotho (*KLB*) expression were undistinguishable between WT and BP3^−/−^ mice (Fig. 1E,F).

We next measured circulating lipids in BP3^−/−^ and WT mice. Circulating phospholipids, non-esterified fatty acids (NEFA), and cholesterol remained unchanged when comparing the genotypes (Fig. 1G). However, BP3^−/−^ mice displayed significantly lower baseline TG levels in agreement with findings of KOMP (Fig. 1G & Supplemental Fig. 1D). To gain further insights into BP3-induced metabolic alterations, we performed comprehensive, unbiased serum and liver metabolomics analyses in WT and BP3^−/−^ mice. In line with decreased serum triglycerides, we found a striking ~14-fold reduction of 9,10,13-trihydroxystearic acid serum levels in BP3^−/−^ mice (Fig. 1H). We also detected increased circulating levels of arachidonic acid and 5-HETE, ω-3-polyunsaturated fatty acids (ω-3-PUFAs) precursor and metabolite respectively (Fig. 1H), that have been previously connected with worsened insulin resistance^28^.

Livers from BP3^−/−^ mice were similar in size and macroscopic and microscopic appearance relative to those of WT littermates. In line with the levels of lower circulating TG, the amount of hepatic TG were also two-fold lower in BP3^−/−^ mice relative to WT controls, whereas the total hepatic lipid content was indistinguishable between the different genotypes (Fig. 1I). A liver metabolomics analysis showed a >2-fold increase of hepatic alpha-linolenic acid, an ω-3-PUFA precursor (Fig. 1J) and, most prominently, a 4.5- to 40-fold reduction of different TG isomers in BP3^−/−^ mice (Fig. 1K). Thus, the loss of BP3 impacts hepatic accumulation of TGs, but not total lipids.

To evaluate the underlying pathways, we measured changes in baseline expression of key fatty acid and TG synthesis enzymes and their upstream regulators in both livers and WAT of BP3^−/−^ and WT mice (Fig. 1L,M). Interestingly, *Ppargc1b*, a key upstream regulator of lipogenesis and steatosis^29^, was significantly reduced in BP3^−/−^ mice. As a consequence, essential de novo lipogenesis enzyme genes such as acetoacetyl-coenzyme A synthetase (*Aacs*), acetyl-CoA carboxylase Alpha (*Acac*), ATP citrate lyase (*Acly*), acetyl-coenzyme A synthetase 2 (*Acas2*), and fatty acid synthase (*Fasn*) were significantly downregulated in BP3^−/−^ animals. Moreover, the lack of BP3 reduced expression of Stearoyl-CoA desaturase 1 (*Scd1*) and Diacylglycerol O-acyltransferase 1 and 2 (*Dgat1* and *Dgat2*), that are rate limiting enzymes for TG synthesis^30^ (Fig. 1L). Silencing of the lipogenic enzymatic machinery in mice lacking BP3 provides an explanation for the reduced serum stearic acid and TG levels. The effect appears to be liver specific and was not seen in the comparison of WAT gene expression (Fig. 1M). These data altogether suggest that endogenous BP3 controls glucose and lipid metabolic pathways.

#### Chronic expression of BP3 in ob/ob mice reduces hyperglycemia, weight gain, steatosis, WAT and BAT mass and liver lipids

**Figure 2:**
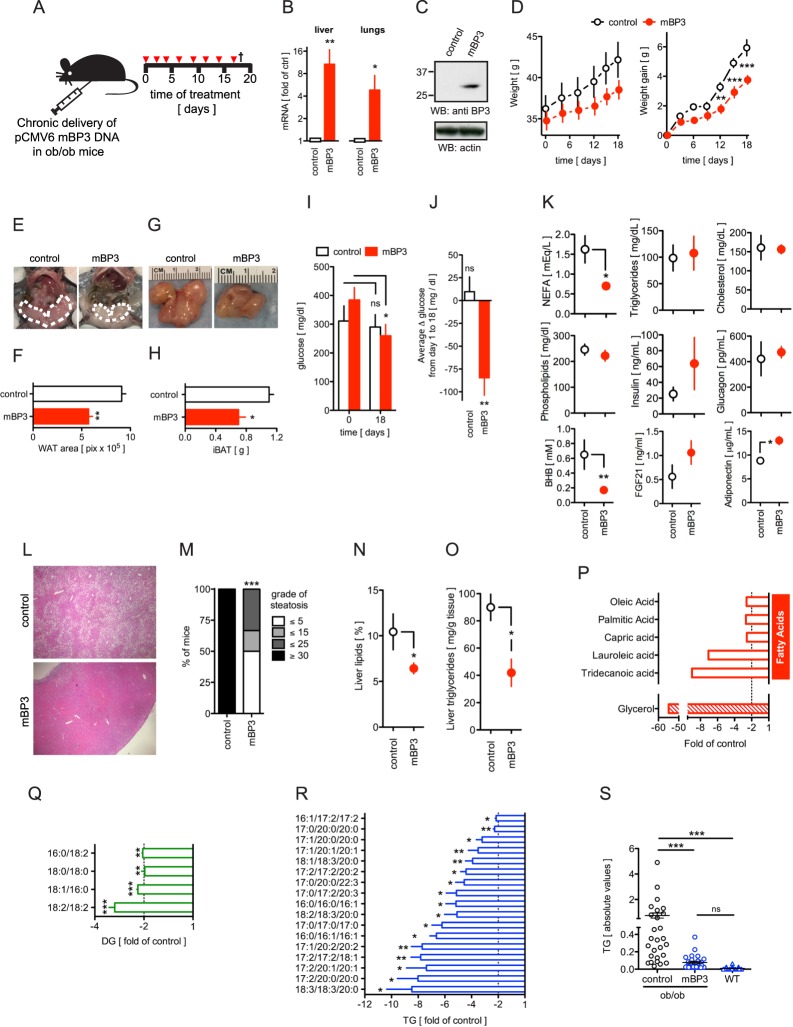
Chronic BP3 expression in ob/ob mice by *in vivo* transfection reduces body weight, WAT, BAT and hepatic steatosis. (**A**) Schematic diagram showing the protocol for the *in vivo* transfection with a murine BP3 (mBP3) expression vector in ob/ob mice. Mice received eight treatments and were euthanized 18 days after the first treatment. (**B**) mBP3 mRNA expression in livers and lungs from transfected ob/ob mice determined by qRT-PCR. Values are shown as fold of control. (**C**) Western blot analysis for mBP3 protein in lungs. (**D**) Total body weight and weight gain of control and mBP3 transfected ob/ob mice. Data were analyzed with two-way ANOVA followed by Bonferroni post tests. (**E**) Representative photographic images of viscera. The white dotted lines indicate visceral WAT. (**F**) Quantitation of the area occupied by visceral fat. (**G**) Representative macroscopic photographs of iBAT. (**H**) iBAT weight. (**I**) Random blood glucose in fed control and mBP3 transfected ob/ob mice before treatment and at the end of the experiment (day 18). (**J**) Average Δ glucose in fed control and mBP3 transfected ob/ob mice throughout the course of the experiment. (**K**) Concentrations of serum metabolites in control and mBP3 transfected ob/ob mice. (**L**) Representative 4x H&E-stained sections of livers. (**M**) Semiquantitative scoring of hepatosteatosis from H&E-stained sections of livers. ***P < 0.0001, χ^2^ test. (**N**) Hepatic lipids and **(O**) TG levels in control and mBP3 transfected ob/ob mice. (**P–R**) Liver metabolomics analysis of (**P**) Glycerol and fatty acids; (**Q**) Diacylglycerides (DG) and (**R**) TG isomers in mBP3 transfected ob/ob mice (fold of control). (**S**) Liver triglyceride levels in control and mBP3 expressing ob/ob and lean C57BL/6 J (WT) mice. All values are expressed as mean ± SEM, n = 3 (control); 6 (mBP3 transfected). ns, non significant; *P < 0.05; **P < 0.01; ***P < 0.0001.

We next sought to identify BP3 mechanisms of action in the modulation of lipid and glucose metabolism. For this, we chronically administered a BP3 expression vector to leptin deficient ob/ob mice, an animal model with metabolic disease and dysregulated lipogenic and gluconeogenic genes^31–33^. We delivered a plasmid that expressed full length murine BP3 (=mBP3) repeatedly over a period of 18 days using a cationic polymer carrier (Fig. 2A) and detected exogenous mBP3 mRNA in livers and lungs by qPCR (Fig. 2B) as well as protein in lungs by immunoblot analysis (Fig. 2C). We transduced the plasmid repeatedly as mice showed a progressive loss of exogenous mBP3 in the liver as early as 6 days after treatment (Supplemental Fig. 2). The mBP3 expressing ob/ob mice exhibited significantly lower body weight and weight gain relative to control littermates (Fig. 2D), paralleled by a reduction of visceral white adipose tissues (WAT) (Fig. 2E,F) and interscapular brown adipose tissues (iBAT) (Fig. 2G,H). A crude measurement of food intake performed in two independent experiments indicated a slight increase of food consumption in mBP3 treated cohort [3.03 ± 0.38 vs. 3.09 ± 0.17 g/day (n = 3–6), and 3.86 ± 0.49 vs. 4.60 ± 0.50 g/day; ctrl vs. mBP3, (n = 5)] that was not statistically significant, indicating that BP3 does not suppress appetite. Administration of mBP3 significantly reduced hyperglycemia (−124.5 ± 58.2 mg/dl glucose at day 18 vs day 0) (Fig. 2I) whereas blood glucose levels in control ob/ob animals remained unaltered (−21 ± 68 mg/dl glucose at day 18 vs day 0) (Fig. 2I). Moreover, during the course of the study, mean daily blood glucose was significantly lowered in the mBP3 group (−84.58 ± 47.03 vs 9.67 ± 39.87 mg/dl glucose; mBP3 vs control) (Fig. 2J). The amelioration of hyperglycemic conditions in mBP3 overexpressing ob/ob mice was parallel by a significant reduction of BHB (Fig. 2K). Furthermore, when compared to control ob/ob littermates, exogenous mBP3 expressing mice exhibited a significant decrease of serum non-esterified fatty acids (NEFA) and increase of FGF21 and adiponectin, whereas TG, cholesterol, phospholipids and glucagon levels remained unchanged (Fig. 2K). It has been shown that increased circulating NEFA levels in ob/ob mice contribute to the onset of hepatic macro- and microsteatosis^34,35^, whereas adiponectin, an FGF21-induced adipokine, is negatively correlated to the hepatic fat content^36^. Strikingly, commensurate with the reduced NEFA and increased FGF21 and adiponectin levels in the serum, livers of mBP3 expressing ob/ob mice displayed a significant reduction of steatosis and were almost indistinguishable microscopically from healthy livers (Fig. 2L,M). Chronic mBP3 treatment induced a slight reduction of liver gross weight (2.43 ± 0.12 vs 2.06 ± 0.09, ctrl vs mBP3) and failed to cause hepatocyte proliferation as measured by *ex vivo* BrdU incorporation (Supplemental Fig. 3). As a consequence of liver remodeling, we observed an increased number of proliferating liver sinusoidal cells. Commensurate with the amelioration of steatosis, mBP3 expressing ob/ob mice exhibited a significant decrease of total liver lipids (Fig. 2N) and triglycerides (Fig. 2O) when compared to ob/ob control animals. Hepatic metabolomics profiling of mBP3 transduced mice showed a 55-fold reduction of glycerol and between 2.5 to 9-fold decrease of the oleic, palmitic, capric, lauroleic and tridecanoid NEFAs (Fig. 2P), that resulted in a significant decrease of hepatic diglycerides (DG) (Fig. 2Q) and TG (Fig. 2R), particularly those containing palmitic (16:0), stearic (18:0) and arachidic (20:0) acids. Interestingly, the amount of hepatic TG in mBP3 treated animals was blunted to levels undistinguishable from those of WT lean mice (Fig. 2S).

In summary, these data indicate that sustained BP3 treatment in ob/ob mice reduces body weight, hyperglycemia, fat mass and steatosis likely due to an increase of serum adiponectin and reduction of circulating NEFA and intrahepatic lipids and without inducing a hepatocellular mitogenic response.

#### Chronic expression of BP3 in ob/ob mice suppresses gluconeogenic and lipogenic gene expression

**Figure 3:**
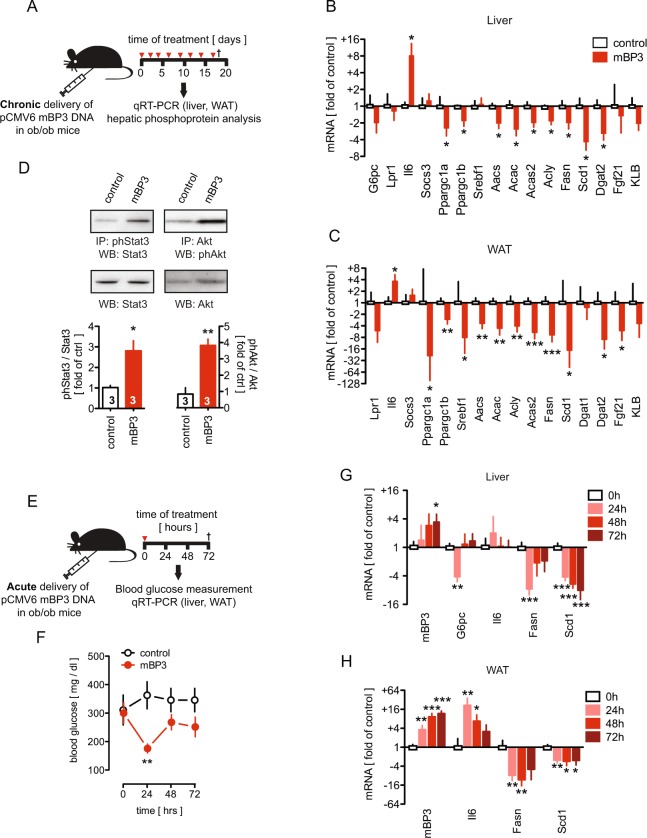
Chronic BP3 expression induces hepatic Stat3 and Akt activation and modulates glucose and fat enzymatic cascades in ob/ob mouse livers and WAT. (**A**) Schematic diagram showing the protocol for the *in vivo* transfection of an mBP3 expression vector in ob/ob mice. (**B**,**C**) Changes in liver (**B**) and WAT (**C**) gene expression analyzed by qRT-PCR in mBP3 transfected ob/ob mice (n = 6). Values are calculated as fold of control (n = 3). (**D**) Stat3 and Akt phosphorylation in livers from mBP3 transfected ob/ob mice or control (n = 3) analyzed by immunoprecipitation (IP) and western blot (WB). Fold activation over controls is shown. (**E**) Schematic diagram showing the *in vivo* delivery of a single dose of an mBP3 expression vector in ob/ob mice. Mice were euthanized 72 hours post treatment. (**F**) Random blood glucose levels at 0, 24, 48 and 72 hours post treatment in control and mBP3 transfected fed ob/ob mice. (**G**,**H**) Change in gene expression in ob/ob mouse livers (**G**) and WAT (**H**) measured by qRT-PCR at the times indicated after a single delivery of mBP3 or control DNA (n = 4). Data are expressed as mean ± SEM. *P < 0.05; **P < 0.01; ***P < 0.0001.

We next examined hepatic and WAT gene expression changes in ob/ob mice after chronic treatment with the BP3 expression vector (Fig. 3A). Consistent with a reduction of blood glucose (Fig. 2I,J), mBP3 overexpression resulted in a two-fold suppression of glucose-6-phosphatase (*G6pc*), the hepatic rate-limiting enzyme for gluconeogenesis. This is likely mediated by the increase of interleukin 6 (*Il6*) and a decrease of peroxisome proliferator-activated receptor-γ coactivator-1α (*Ppargc1a*) expression (Fig. 3B). *Il6* and *Ppargc1a* were similarly regulated in WAT (Fig. 3C). Notably, no changes of hepatic *Lpr1*, *Socs3*, *Fgf21 or KLB* mRNA expression were observed after chronic BP3 administration (Fig. 3B,C). Next, we evaluated the regulation of the *de novo* lipogenesis genes in both liver and WAT of mBP3 transduced ob/ob mice. Expression of the lipogenesis regulator *Fgf21* was downregulated in WAT whereas *Ppargc1b* and *Srebf1*^29,37^ were significantly reduced in both tissues, likely resulting in the observed downstream suppression of *Aacs*, *Acac*, *Acas2*, *Acly*, and *Fasn*. Additionally, *Scd1* and *Dgat2* were significantly reduced in both livers and WAT (Fig. 3B,C). Finally, uncoupling protein 1 (*Ucp1*) mRNA levels in the BAT were indistinguishable between the BP3 treated and the control group indicating no obvious effect on thermogenesis regulation (Supplemental Fig. 4).

To investigate the effect of a chronic mBP3 administration on signaling pathways that govern carbohydrate and fatty acid metabolism, we analyzed the phosphorylation state of Akt and Stat3, key effector molecules of gluconeogenic and lipogenic genes in ob/ob mice^38,39^. Sustained mBP3 expression increased hepatic endogenous Stat3 and Akt phosphorylation by 2.8 and 3.9 fold, respectively, while total Stat3 and Akt proteins remained unchanged (Fig. 3D).

A single administration of the mBP3 expression vector to *ad libitum* fed ob/ob mice (Fig. 3E) transiently reduced the pathologically elevated blood glucose levels of >300 mg/dl significantly to normoglycemia (Fig. 3F), whereas control mice remained hyperglycemic. This effect was corroborated by a transient four-fold downregulation of hepatic *G6pc* mRNA expression (Fig. 3G) and was paralleled by a significant mBP3 overexpression in both liver and WAT up to 72 hours post transduction (Fig. 3G,H). Moreover, a sustained suppression of *Fasn* and *Scd1* mRNAs in livers and WAT was observed whereas upregulation of *Il6* was seen exclusively in the WAT for 24, 48 and 72 hours post transduction (Fig. 3G,H).

Overall, our data suggest that chronic BP3 treatment in ob/ob mice results in the suppression of rate-limiting genes regulating metabolic pathways in the liver and WAT that control the synthesis of lipids and glucose.

#### Chronic expression of BP3 in diet-induced obesity (DIO) mice suppresses hyperglycemia, weight gain and hepatic and WAT lipogenic gene expression

**Figure 4:**
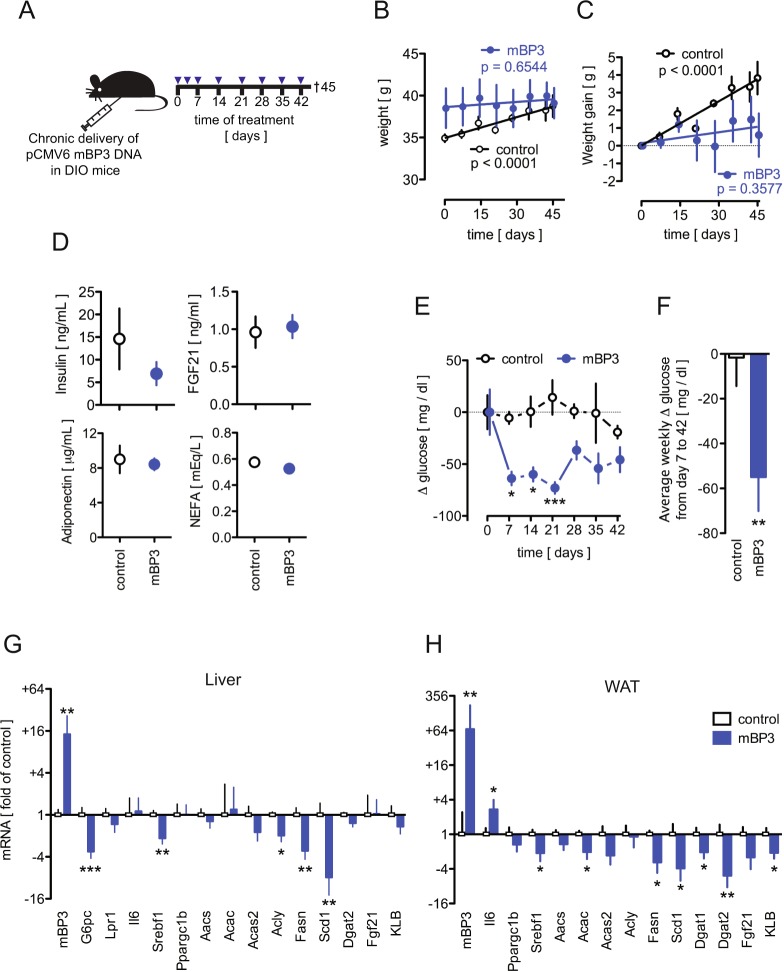
Chronic BP3 expression in DIO mice reduces body weight, hyperglycemia and modulates lipogenic gene expression in liver and WAT. (**A**) Schematic diagram showing the protocol for the *in vivo* transfection of mBP3 or control expression vector in C57BL/6 J DIO mice. Mice received eight treatments and were euthanized 45 days after the first treatment. (**B**,**C**) Total body weight (**B**) and body weight gain (**C**) of control and mBP3 transfected DIO mice, analyzed by linear regression. (**D**) Concentrations of serum metabolites in control and mBP3 transfected DIO mice. (**E**) Random blood glucose in fed control and mBP3 transfected DIO mice measured every 7 days. (**F**) Average change in blood glucose in fed control and mBP3 transfected DIO mice throughout the course of the experiment. (**G**,**H**) Change in gene expression in DIO mouse livers (**G**) and WAT (**H**) measured by qRT-PCR. Data are expressed as fold of control (Mean ± SEM; n = 5; *P < 0.05; **P < 0.01; ***P < 0.0001).

Next, we sought to investigate the response to exogenous mBP3 in C57BL/6J DIO (=diet-induced obesity) mice, an animal model that mimics the most common cause of human obesity^40^. mBP3 plasmid or an empty vector control were administered eight times over a period of 42 days to 14 week-old DIO mice (Fig. 4A). Consistent with our findings in the ob/ob mouse model (Fig. 2), BP3 exhibited a significant anti-obesity and antidiabetogenic effect. mBP3 treated DIO mice, in contrast to their control littermates, failed to gain body weight, despite a high calorie intake (Fig. 4B,C). Circulating insulin, FGF21, adiponectin, and NEFA remained unchanged between the two groups (Fig. 4D). Still, mBP3 treated DIO mice exhibited significantly lower blood glucose (Fig. 4E,F) that coincided with a significant 3.5 fold reduction of hepatic *G6pc* mRNA (Fig. 4G). Moreover, BP3 treatment induced a significant downregulation of hepatic *Srebf1* mRNA and its target genes, such as *Acly*, *Acas2*, *Fasn* and *Scd1*. However, *Lepr*, *Il6*, *Ppargc1b*, *Acac*, *Dgat2*, *Fgf21* and *KLB* transcript level remained unchanged from the control (Fig. 4G). Similarly, WAT *Fgf21*, *KLB and Srebf1* mRNA levels were reduced as well as *Acac*, *Acas2*, *Fasn*, *Scd1*, *Dgat1* and *Dgat2*, but not *Ppargc1b*, *Aacs*, or *Acly* (Fig. 4H).

Taken together these data suggest that BP3 impacts the de novo lipogenesis and TG synthesis enzymatic cascade also in the DIO mouse model.

### BP3 binds to endocrine FGFs and enhances their signaling *in vitro*

**Figure 5:**
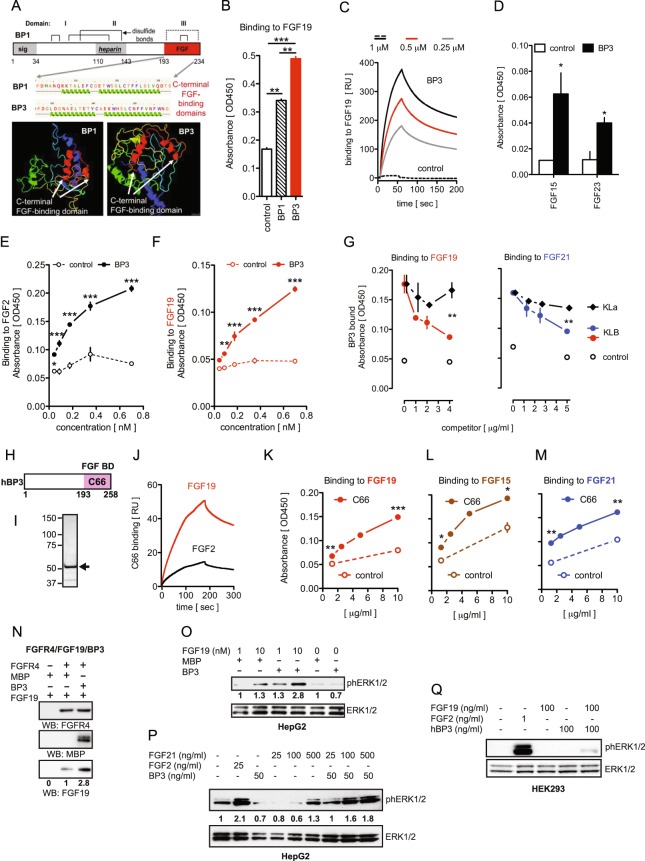
BP3 binds to endocrine FGFs, enhances FGF19 and FGF21 signal transduction and promotes FGFR4/FGF19 complex formation. (**A**) Domains and computational structure prediction for BP1 and BP3. The FGF-binding domains in BP1 and BP3 in the C-terminal portion, the heparin-binding domain and the conserved cysteins of BP1 are shown. The white arrows in the 3D model indicate distinct folding of the FGF-binding domains. The PHYRE2 program (http://www.sbg.bio.ic.ac.uk/phyre/) was used^68^. (**B**) Binding of MBP-tagged BP1 or BP3 or MBP control to immobilized FGF19 measured by direct ELISA with an anti-MBP antibody. Mean ± SEM of one of three independent experiments done in duplicate (**P < 0.01; ***P < 0.0001). (**C**) SPR sensorgrams illustrating the binding of BP3 to immobilized FGF19. The concentrations of the BP3 analyte are indicated. RU, response units. (**D**) Binding of BP3 or MBP control to immobilized FGF15, or FGF23 measured by direct ELISA with an anti-MBP antibody. Mean ± SEM of one of three independent experiments done in duplicate. *P < 0.05 BP3 (black bars) vs. control (white bars). (**E**,**F**) Equilibrium binding of BP3 to immobilized FGF2 (**E**) or FGF19 (**F**) measured by direct ELISA with an anti-MBP antibody. Mean ± SEM of one of three independent experiments done in duplicate (**P < 0.01; ***P < 0.0001; Two-way ANOVA). (**G**) Competition of KLB and KLa proteins for binding of BP3 to immobilized FGF19 (left panel) or FGF21 (right panel). Mean ± SEM of one of three independent experiments run in duplicate (*P < 0.05; **P < 0.01 BP3 + KLB (red or blue dot) vs. BP3 + KLa (black diamond); Two-way ANOVA). (**H**) Schematic representation of human BP3. The numbers correspond to the human BP3 amino acid sequence. (**I**) Coomassie blue staining of MBP-tagged C66 fusion protein purified by amylose affinity chromatography. The arrow indicates a band of an apparent molecular mass of 52 kDa. (**J**) SPR sensorgrams illustrating the binding of C66 to immobilized FGF19 and FGF2. RU, response units. (**K**,**L**,**M**) Equilibrium binding of C66 to immobilized FGF19 (**K**), FGF15 (**L**) or FGF21 (**M**) measured by direct ELISA with an anti-MBP antibody. Mean ± SEM of one of two independent experiments done in duplicate (*P < 0.05**; P < 0.01; ***P < 0.0001; t-test). (**N**) Detection of FGFR4-FGF19-BP3 protein complex. *In vitro* binding of FGF19 ± BP3 to immobilized FGFR4 was detected by western blot analysis. The numbers below the blots indicate the fold-change of FGF19 corrected for FGFR4. Samples derive from the same experiment and gels/blots were processed in the same gel. The blots are representative of three independent experiments. (**O**,**P**) Phospho and total ERK1/2 in HepG2 cells treated for 15 minutes with different concentrations of FGF19 ± BP3 (**O**) or FGF21 ± BP3 (**P**). FGF2 was used as a positive control for ERK1/2 activation. Controls were treated with MBP. The numbers below the blots indicate the fold-change, corrected for total ERK1/2. (**Q**) Phospho and total ERK1/2 in KLB-negative HEK293 cells treated for 15 minutes with FGF19 ± BP3 (100 ng/ml). FGF2 was used as a positive control for ERK1/2 activation.

The interactions of FGF-binding proteins (BPs) with heparin-binding, paracrine FGFs have been well-defined. BP1 was found to supplement for a lack of HS in FGF2 signaling^1,2,4,5^. From this we expected a different interaction of BPs and endocrine FGFs that exhibit a low heparin affinity [reviewed in^41^]. The predicted structures of the BP1 and BP3 C-terminal FGF binding domains^2,4,5,42^ show distinct features (Fig. 5A) and we thus compared the interaction of both proteins with FGF19, the prototypic member of the endocrine FGF family. We generated an MBP-tagged human recombinant BP3 devoid of its signal peptide (=BP3), purified it by affinity chromatography (Supplemental Fig. 5A) and verified its purity and identity by Coomassie blue staining and western blot analysis with anti-hBP3 and MBP antibodies (Supplemental Fig. 5B,C) as well as mass spectrometry (Supplemental Fig. 5D). Surprisingly, FGF19 showed a significantly stronger binding to BP3 than to BP1 (Fig. 5B). Binding of BP3 to FGF19 was also confirmed by surface plasmon resonance (SPR; Biacore) (Fig. 5C). BP3 bound with analogous affinity to FGF15, the FGF19 murine ortholog, and to FGF23 (Fig. 5D). Moreover, as shown in a solid-phase equilibrium binding assay, BP3 could bind to FGF2 and FGF19 in a similar fashion, with a Kd = 0.269 ± 0.273 nM and Kd = 0.172 ± 0.341 nM, respectively (Fig. 5E,F).

In support of the notion that FGF19 and FGF21 utilize KLB as co-receptor^41^, we sought to investigate the effect of BP3 on the FGF/KLB interaction. Excess concentrations of KLB, but not α-Klotho (KLa), inhibited the interaction of BP3 with either FGF19 or FGF21 (Fig. 5G) indicating mutually exclusive binding of either BP3 or KLB to these endocrine FGFs.

Because endocrine FGFs do not utilize HS, we tested the effects of a C-terminal fragment of the BP3 protein (C66) that lacks the BP3 heparin-binding domain (HBD) (Fig. 5H). MBP-tagged C66 was purified by affinity chromatography and its purity established by Coomassie blue staining (Fig. 5I). By SPR, the C66 fragment displayed a higher binding affinity for FGF19 than for FGF2, which distinguished it from the full-length protein (Fig. 5J). In addition, in a solid-phase equilibrium binding assay, C66 showed binding to increasing concentrations of FGF19, FGF15 and FGF21 (Fig. 5K–M). These data suggest that the C-terminal FGF-binding domain of BP3 is sufficient for the interaction with endocrine FGFs.

Since the preferential receptor of FGF19 is FGFR4^43^, we examined the interaction of BP3 and FGF19 with immobilized recombinant FGFR4 in a cell-free assay. The inclusion of BP3 enhanced FGF19 - FGFR4 binding by 2.8-fold (Fig. 5N) and we conclude that BP3 favors the formation of a stable complex between FGF19 and FGFR4.

FGF19 and FGF21 have been shown to activate ERK1/2 in HepG2 cells^44,45^. We found that addition of BP3 to FGF19 increased the activation of ERK1/2 by 1.3 to 2.8 fold, while BP3 alone did not show an effect (Fig. 5O). Similarly, FGF21-induced ERK1/2 activation was enhanced by added BP3 by ~1.8 fold (Fig. 5P). Klotho deficient-HEK293 cells will signal in response to endocrine FGFs only after KLa or KLB overexpression, whereas FGF2 can induce a robust downstream ERK1/2 activation^46,47^ (and Fig. 5Q). Interestingly, exogenous co-administration of recombinant BP3 and FGF19 proteins to HEK293 cells was sufficient to elicit ERK1/2 phosphorylation, while either protein alone had no detectable effect (Fig. 5Q). This shows that BP3 is sufficient to promote receptor activation by an endocrine FGF in the absence of KLB. We conclude that BP3 binds to the endocrine FGFs, competes with KLB, and can enhance FGF19 and FGF21 signaling *in vitro*.

**Figure 6:**
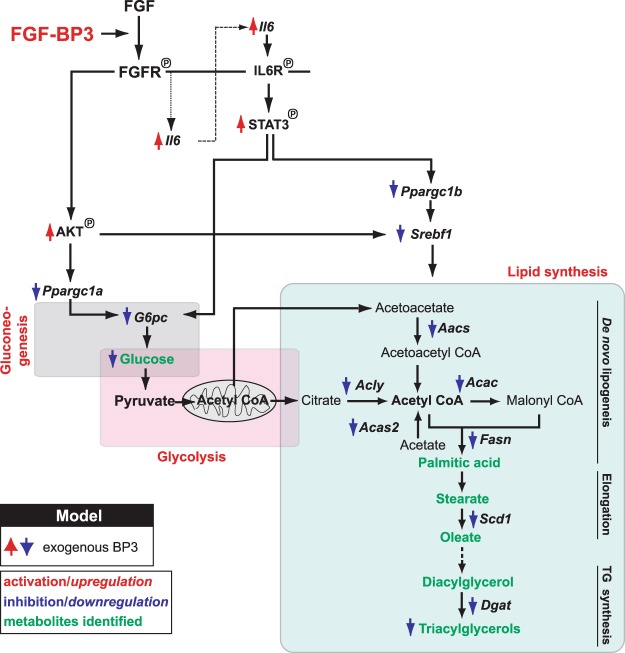
Summary of the pathways impacted by chronic BP3 treatment in liver and WAT in obese mouse models. BP3 enhances FGF/FGFR signaling and downstream activation of Akt and IL6/Stat3 that lead to the inhibition of gluconeogenesis, through *Ppargc1a/G6pc* downregulation. The parallel activation of Akt and Stat3 results in an inhibition of *Ppargc1b* and/or *Srebf1*, key regulators of downstream lipogenic enzymes, thus leading to the inhibition of *de novo* lipogenesis and TG synthesis.

In composite, our data suggest that BP3 is a metabolic modulator that interacts with endocrine FGFs and can ameliorate metabolic syndrome pathology by modulation of glucose and lipid metabolism in liver and adipose tissues. The schematic in Fig. 6 connects the signaling pathways to the respective metabolic enzyme cascade and biological function impacted by chronic BP3 treatment in obesity models.

## Discussion

In this report we show a new role for BP3 in regulating carbohydrate and lipid metabolism that overlaps with the effects of endocrine FGF19 and FGF21^48^. BP1 and BP3 proteins share 8 conserved cysteine residues responsible for the formation of disulfide bonds^4^ but possess a distinct C-terminal FGF binding domain, as evidenced by the secondary structure prediction (see Fig. 5A). We had hypothesized that these structural differences could translate into differential affinity and selectivity of BP1 and BP3 for different FGFs. BP1 and BP3 have been shown to bind to paracrine FGF1 and 2 and modulate their signaling and biological functions^2,4^. ECM-bound paracrine FGFs are released by BPs that compete with the binding of FGF to the heparin-rich ECM^2^. In contrast to the paracrine FGFs, endocrine FGFs are characterized by a weak heparin binding, as they lack the critical GXXXXGXX(T/S) binding motif. This explains their ability to freely circulate and thus act in a systemic, hormone-like, rather than local fashion^41^. A surprising result from our present study is that BP3 binds with high affinity to FGF19, the prototype member of the endocrine FGF family, and to FGF21 and FGF23, the other two members of the family, whilst BP1 exhibits a lower affinity. Notably, the C-terminal FGF-binding domain (C66) exhibits a higher affinity for FGF19 than FGF2. Furthermore, the interaction between FGF19 and BP3 stabilizes the ligand/receptor complex and enhances downstream signaling. Based on the competitive binding of BP3 and KLB proteins to FGF19 and FGF21 and the ability of BP3 to evoke FGF19 signaling *in vitro* despite the lack of KLB, we propose that BP3 may function as a co-receptor for these endocrine FGFs.

The ablation of endogenous BP3 did not impact embryo viability or overall survival but was associated with abnormal GTT and reduced levels of circulating triglycerides, suggesting an increased risk for metabolic disease (Supplemental Fig. 1D). Both FGF19 and FGF21 are insulin-independent modulators of carbohydrate and lipid metabolism in rodents, primates and humans^48^. Knockout mouse models for FGF15, the mouse ortholog of FGF19, and for FGF21 develop glucose intolerance and insulin resistance^49,50^. Similarly, BP3^−/−^ mice showed trends of hyperinsulinemia and upregulation of key hepatic gluconeogenic genes that can result in an increase of fasting blood glucose and abnormal insulin resistance.

BP3 did not seem to be differentially regulated between lean C57BL/6 J and ob/ob or DIO mouse ilea, livers and WAT (Supplemental Fig. 6). However, a survey of published gene expression data in tissues from diabetic human subjects or from diabetes-related mouse models showed a significant downregulation of BP3. BP1 expression was typically not correlated with the disease (Supplemental Fig. 7A–F). This supports a contribution of endogenous BP3 to the mechanisms underlying metabolic disease revealed by the phenotypic characteristics of the BP3^−/−^ mice (see Fig. 1).

Exogenous BP3 induced a robust activation of hepatic STAT3 and inhibition of *Ppargc1b* and *Srebf1*, the transcriptional regulators of lipogenic genes, resulting in reduced de novo lipogenesis, FA elongation and TG synthesis (see scheme in Fig. 6). Chronic expression of BP3 indeed resulted in a dramatic reduction of white and brown fat mass and an amelioration of hepatic steatosis. Mechanisms that induce fatty liver disease are mostly due to increased NEFA delivery from peripheral WAT and a greater de novo lipogenesis alongside with insulin resistance, whereas β-oxidation of FA seems to be of marginal importance^34,37^. That is, chronically treated ob/ob mice exhibit reduced circulating and liver fatty acids and increased circulating FGF21 and adiponectin as well as a significant suppression of the de novo hepatic lipogenic enzymatic cascade, providing an explanation for the reversal of steatosis. It is noteworthy that circulating NEFA levels are reduced in mice after recombinant FGF19 and FGF21 treatment and are elevated in FGF21^−/−^ mice^43,51^. Consistent with our findings, the amelioration of a fatty liver phenotype in FGF21-treated DIO mice results from the suppression of the Srebf1-induced fatty acid biosynthetic pathway^52^. Moreover, the beneficial effects of FGF21 on obesity-associated metabolic disorders are dependent on the increase of adiponectin mRNA and protein and are obliterated in adiponectin-deficient mice^53^. Notably, the effect of BP3 treatment on weight gain mimics the effect of FGF21 in ob/ob and in the DIO mouse models^54^.

The improved glycemic control in obese mice after acute or chronic BP3 treatment was also paralleled by a downregulation of *G6pc*, the rate limiting gluconeogenic enzyme, likely resulting from sustained hepatic Akt and Stat3 activation and Il6 upregulation. We found that BP3 activates the IL6/STAT3 signaling axis, which represents a previously unknown FGF-response pathway. To date, the role of IL6 in obesity-associated insulin resistance is controversial^55^: Impaired insulin action can be induced by increased IL6 levels *in vivo* or vice versa, thus denoting an unfavorable metabolic effect of IL6^56,57^. Conversely, in gain-of-function studies, others have suggested that IL6 can have an anti-inflammatory beneficial role in preventing obesity and T2D in a STAT3-dependent manner^58^. Moreover, IL6 can have an insulin sensitizing effect by increasing glucose uptake in cultured and intact human skeletal muscle^59^ and by modulating insulin release after glucagon-like peptide-1 secretion from intestinal L and pancreatic alpha cells in response to exercise^60^. In our studies, IL6 upregulation, seen after BP3 treatment, matches with improved insulin sensitivity, obesity and steatosis in IL6 transgenic mice^58,61,62^, likely by suppressing gluconeogenic and lipogenic enzymatic cascade via hepatic STAT3 phosphorylation (see scheme in Fig. 6). Complementary to this, hepatocyte-specific IL6R, IL6, or STAT3 deficient mice develop increased gluconeogenesis and insulin resistance, as a result of G6pc upregulation, likely via a PI3K/FoxO1-independent pathway, as well as age-related obesity and steatosis^38,63^.

Obesity, which affects more than 650 million people worldwide, is the major driver for metabolic syndrome, which also encompasses disorders such as insulin resistance, glucose intolerance, hypertension and dyslipidemia^23^. Our studies show that the interaction between BP3 and endocrine FGFs could be leveraged for a novel approach to the treatment of metabolic disease and the associated symptoms. Both FGF19 and FGF21 are potential therapeutic agents for the treatment of metabolic syndrome^48,64^. However, the mitogenic effects of FGF19 and the induction of hepatocellular carcinoma in FGF19 transgenic mice and in mice treated with the recombinant protein are of concern^65^. In contrast, FGF21 lacks a proliferative activity both *in vitro* and *in vivo*^66^. Moreover, in line with these observations, a recent report has proposed that the endocrinization of FGF1 exhibits anti-diabetogenic and non-mitogenic effects in mice^67^. It is noteworthy that when chronically delivered to ob/ob mice, BP3 fails to induce a hepatocellular mitogenic response, thus matching with the lack of mitogenic effects reported for FGF1 and FGF21.

In conclusion, the striking reduction of fat mass and fatty liver disease as well as hyperglycemia and the lack of a mitogenic response make BP3 an attractive candidate for the treatment of metabolic disease.

## Methods

### Enzyme-linked immunosorbent assay (ELISA)

High binding ELISA plates (Nest Scientific, Rahway, NJ) were coated with 100 μl/well of recombinant proteins (human recombinant FGF2, FGF15, FGF19, or FGF23, 7.5 μg/ml] and incubated overnight at 4 °C. Plates were washed thrice between each incubation step with washing buffer [1X Phosphate buffered saline with 0.2% Tween 20, pH 7.4 (PBST)]. Blocking was carried out with 100 μl/well of 5% dry milk diluted in PBST for 1 hour at room temperature. Subsequently, plates were incubated for 2 hours at room temperature with 100 μl/well of an MBP-tagged recombinant protein (MBP control, BP3 or C66) at a fixed concentration (0.7 nM) or in serial dilutions. Detection was carried out with 100 μl/well of an anti-MBP mouse monoclonal antibody (New England BioLabs, Ipswich, MA) and with an affinity-purified goat anti-mouse horseradish peroxidase (HRP)-conjugated antibody (GE Healthcare Life Sciences, Pittsburgh, PA) (1:1,000 dilution in PBS). The reactions were visualized with the aid of 1-Step Turbo TMB (Thermo Scientific, Grand Island, NY), according to the manufacturer’s protocol, and read with an Ultramark Microplate Imaging System (Bio-Rad Laboratories, Hercules, CA) at 450 nm absorbance. For the competitive protein binding assays, increasing excess amounts of β-Klotho (KLB) or α-Klotho (KLa) (competitors) were bound to immobilized FGF19 or FGF21 (2.5 μg/ml) for 1 hour prior to the addition of BP3 or MBP control (0.15 μg/ml) in MaxiSorp microtiter plates (Sigma Aldrich, St. Louis, MO). Human recombinant FGF2 was purchased from Life Technologies (Grand Island, NY). Recombinant human FGF21, human FGF23, human KLa and mouse KLB were purchased from R&D Systems (Minneapolis, MN). Recombinant FGF15 was purchased from MyBioSource (San Diego, CA). Human recombinant FGF19 was generated in the lab of Dr. Moosa Mohammadi at NYU.

#### Surface Plasmon Resonance Binding Assay

Biacore T200 instrument (GE Healthcare, Piscataway, NJ) was used for surface plasmon resonance measurements. Human recombinant FGF19 or FGF2 in HBS-P buffer (0.01 M HEPES pH 7.4, 0.15 M NaCl, 0.05% P-20) were immobilized on a flow cell of a CM-5 sensor chip (GE Healthcare) via amine coupling. A blank flow cell was used as a negative control for non-specific binding to the sensor surface. BP3 (1, 0.5, or 0.25 μM) or C66 (245 nM) in HBS-P buffer were injected over the immobilized proteins with a flow rate of 10 µL/min for 60 seconds and the resulting maximum responses were obtained. The experiments were performed in triplicate.

#### Binding of BP3 to FGFR4/FGF19 complex

MaxiSorp microtiter plates were coated with 0.75 mg of human recombinant FGFR4 Fc Chimera (R&D Systems) and incubated overnight at 4 °C. Plates were washed thrice between each incubation step with PBS. Blocking was carried out with 100 μl/well of 5% dry milk diluted in PBS for 1 hour at room temperature. Subsequently, plates were incubated for 2 hours at room temperature with 100 μl/well of FGF19 (2 μg/ml) ± BP3 or MBP control (1 μg/ml). Bound proteins were detected by western blot analysis with 1 μg/ml of an anti FGFR4 (LD1; a kind gift of Genentech, South San Francisco, CA), anti MBP (New England BioLabs) or anti FGF19 (Abnova, Walnut, CA) mouse monoclonal antibodies.

### Cell cultures

Human hepatocellular carcinoma (HepG2) and HEK293 cells were maintained in Dulbecco’s modified Eagle’s medium (Life Technologies) supplemented with 10% (v/v) fetal bovine serum.

### Animals, genotyping and animal treatments

Animal experiments were reviewed and approved by the Institutional Animal Care and Use Committee of Georgetown University. All experiments were performed in accordance with relevant guidelines and regulations. BP3^−/−^ mice were generated by the trans-NIH Knock-Out Mouse Project (KOMP) and obtained from the KOMP Repository (University of California, Davis, CA) (www.komp.org). BP3^−/−^ mice were genotyped by q-PCR (see below) under the following conditions: 95 °C for 3 minutes, followed by 40 cycles (95 °C for 20 seconds, 65 °C for 30 seconds, and 72 °C for 40 seconds). The PCR primers are in Table S1.

Twelve week-old C57BL/6J DIO male mice and six week-old ob/ob female mice were purchased from The Jackson Laboratory (Bar Harbor, ME). C57BL/6 J DIO mice were maintained on a HFD containing 60% kCal from fat (D12492; Research Diets, Inc., New Brunswick, NJ) from weaning for at least 14 weeks before randomization by weight and treatment and until the completion of the study. Body weight ranged from 30 to 47 grams. Animals were maintained in a normal light-cycle room and were provided with regular diet or HFD and water *ad libitum*. Body weight gain was calculated as the daily body weight minus body weight before treatment for each animal and presented as an average for the group.

pCMV6-Kan/Neo mouse FGFBP3 (mBP3) or the empty vector (Origene, Rockville, MD) were delivered *in vivo* by an intraperitoneal (i.p.) injection with the TurboFect *in vivo* Transfection Reagent (ThermoScientific), according to the manufacturer’s instructions. This reagent, developed by Dharmacon, was designed to avoid an inflammatory response in mice as determined by TNFalpha induction by ELISA and also for intraperitoneal injections (http://dharmacon.gelifesciences.com/transfection/turbofect-in-vivo-transfection-reagent/). The endotoxin measure in our plasmid prep was 0.2 EU/ug and thus considered endotoxin free.

### Glucose, insulin and pyruvate tolerance test

Glucose, insulin and pyruvate tolerance tests (GTT, ITT and PTT) were performed by an i.p. injection of glucose (1 g kg^−1^), insulin (0.75 IU kg^−1^) (Novo Nordisk Novolin-R, ADW Diabetes, Pompano Beach, FL) or pyruvate (1 g kg^−1^) (Sigma Aldrich) in mice fasted overnight (for GTT and PTT) or for 6 hours (for ITT). Tail blood glucose concentrations were measured with a portable glucose meter (Contour, Bayer, Whippany, NJ) as indicated in the respective figures. Average Δ glucose was calculated as the group average of daily or weekly blood glucose concentration minus blood glucose concentration before treatment for each animal.

### Histological Analysis

Histologic hepatosteatosis score was performed semiquantitatively on five to 10 H&E-stained paraffin-embedded liver sections by two blinded investigators and expressed as percentage of fatty hepatocytes occupying the hepatic parenchyma.

### Western Blots and Immunoprecipitation

Immunoprecipitation and western blot analyses were performed as described earlier^17^. Briefly, livers or lungs from ob/ob mice were homogenized in 1 mL of lysis buffer with a MagNa lyser homogenizer (Roche, Indianapolis, IN). 5 mg of total liver lysates were immunoprecipitated with 10 μl of sepharose-conjugated anti-Akt or phospho Stat3 antibodies and immunoblotted with an anti-phospho Akt or anti-Stat3 rabbit polyclonal antibodies, respectively. Total Akt and Stat3 in the liver lysates (50 μg) were detected using an anti-Akt and Stat3 rabbit polyclonal antibodies, respectively. ERK1/2 phosphorylation studies in HepG2 cells were performed by immunoblotting for phospho ERK1/2 and anti-ERK1/2 with rabbit polyclonal antibodies, as described^2^. All antibodies were purchased from Cell Signaling (Danvers, MA). mBP3 expression in transfected ob/ob mouse lungs was detected by immunoblot analysis using an anti mouse FGFBP3 rabbit polyclonal antibody (Abgent).

### RNA isolation, microarray analysis and quantitative real-time PCR

Total RNA was isolated from mouse livers, lungs, WAT or ilea using RNeasy Mini kit or RNeasy Lipid Tissue kit (Qiagen, Valencia, CA), according to the manufacturer’s instructions. cDNA was synthesized from 1 μg of total RNA using the iScript cDNA Synthesis Kit, according to the manufacturer’s protocol (Bio-Rad Laboratories, Hercules, CA). Quantitative real-time PCR (qRT-PCR) was performed in a Realplex^2^ (Eppendorf, Hauppauge, NY) using the iQ SYBR Green Supermix (Bio-Rad Laboratories) under the following conditions: 95 °C for 3 minutes, followed by 40 cycles (95 °C for 20 seconds, 60 °C for 30 seconds, and 72 °C for 40 seconds). mRNA levels, determined by qRT-PCR, were normalized to endogenous β-actin mRNA and shown as fold of control. The forward (FW) and reverse (Rev) PCR primers for murine genes used are provided in Table S1.

### Data Analysis and Statistics

GraphPad Prism (La Jolla, CA) was used to compare the means of two or more groups by Student’s *t*-test or ANOVA respectively. Semiquantitative scoring of hepatosteatosis was analyzed by χ^2^ test for trend. Statistical significance was defined as *P* < 0.05.

## Electronic supplementary material

Supplementary information
