# Supplementary material for: Fibroblast Growth Factor Binding Protein 3 (FGFBP3) impacts carbohydrate and lipid metabolism
Source: Sci Rep. 2018 Oct 29;8:15973. doi: 10.1038/s41598-018-34238-5 (PMC6206164; doi:10.1038/s41598-018-34238-5)
Supplement: Supplementary file 1 — Supplementary information [file 41598_2018_34238_MOESM1_ESM.pdf]

Supplemental Material for

Fibroblast Growth Factor Binding Protein 3 (FGFBP3) impacts carbohydrate and lipid metabolism

Elena Tassi, Khalid A. Garman, Marcel O. Schmidt, Xiaoting Ma,

Khaled W. Kabbara, Aykut Uren, York Tomita, Regina Goetz, Moosa Mohammadi,

Christopher S. Wilcox, Anna T. Riegel, Mattias Carlstrom, and Anton Wellstein*

*Correspondence to: wellstea@georgetown.edu

This PDF file includes:

Methods.

Supplemental Figure 1. Generation of BP3$^{-/-}$ mice.

Supplemental Figure 2. Time course of expression of mBP3 after a single administration in ob/ob mice.

Supplemental Figure 3. Chronic mBP3 administration in ob/ob mice does not cause a mitogenic effect in liver parenchyma.

Supplemental Figure 4. Chronic mBP3 administration in ob/ob mice does not regulate Ucp1 in WAT or BAT.

Supplemental Figure 5. Purification and detection of recombinant hBP3.

Supplemental Figure 6. BP3 mRNA expression is not different between control and obese mice.

Supplemental Figure 7**.** BP3 mRNA expression is reduced in diabetic patients and in murine diabetic models.

Supplemental Table 1. qPCR primer list

References.

Full-size Western blots

METHODS

Expression and purification of recombinant hBP3 and C66.

Human BP3 cDNA, correspondent to amino acids 27-258, and the C-terminal hBP3 region, corresponding to amino acids 167-232, were subcloned into a pMAL-p2X vector (New England BioLabs, Ipswich, MA) and MBP-tagged recombinant proteins (hBP3 and C66, respectively) were generated as previously described (1). MBP and MBP-BP3 are referred to as “control” and “BP3”, respectively, throughout the manuscript. Recombinant proteins were purified by fast protein liquid chromatography (FPLC) as described (2). Briefly, bacterial cell lysates were loaded onto an MBPTrap™ HP column (Dextrin Sepharose) (GE Healthcare Life Sciences, Piscataway, NJ) and MBP-tagged proteins eluted with 20 ml of a gradient of 0-10 mM Maltose in Column Buffer (20mM Tris HCl pH 7.4, 200 mM NaCl, 1mM EDTA). Positive fractions were then loaded onto HiTrap Heparin HP columns (GE Healthcare Life Sciences) and proteins eluted with 20 ml of a gradient of 0-1.5M NaCl in Column Buffer. Eluted proteins were analyzed by immunoblotting with an anti hBP3 rabbit polyclonal antibody (Abgent, San Diego, CA) or with an anti MBP mouse monoclonal antibody (New England BioLabs).

Protein Identification by Mass Spectrometry.

Eluted hBP3 was resolved on a 4-12% Bis-Tris gel (Life Technologies, Carlsbad, CA), visualized by Coomassie Blue staining and the bands excised from the gel. Mass spectrometry analysis was conducted as described previously (2).

Metabolomic analysis using UPLC-ESI-QTOF-MS

Samples were processed by initial sonication in extraction solvent containing water and methanol (50% v/v) and internal standards were processed as previously described (3, 4).

The UPLC-QTOF raw data files were converted into NetCDF format (Network Common Data Form) using the MassLynx software (Waters Corporation, Milford, MA). Subsequently, the LC-MS data were preprocessed using XCMS software as described (5).

GC-MS Analysis

A. Sample Preparation: The dried sample extracts were resuspended in 0.1 ml of 1:1 MeOH/ddH_2O (Thermo Fisher) containing 4 μg/mL 4-nitrobenzoic acid (Sigma Aldrich), vortexed and centrifuged. Each mixture was transferred to a separate GC vial containing a 250 μl glass insert (Gerstel, Inc., Linthicum Height, MD). A pooled sample QC was prepared by combining 5 μl of each sample and partitioned into 100 μl aliquots. Moisture was eliminated by drying samples in the Savant Intergrated SpeedVac system ISS 100 (Thermo Fisher, Waltham, MA). For derivatization, each sample vial was administered 40 μl of methoxylamine (MOX, 20 mg/ml) (Sigma Aldrich) and incubated for 50 min at 50°C in the Gerstel Multipurpose Sampler (MPS) agitator. For silylation, each sample vial was administered 40 μl of N-Methyl-N-(trimethylsilyl)trifluoroacetamide (MSTFA) with 1% trimethylchlorosilane (TMS) (Sigma Aldrich) and incubated for 60 min at 50°C in MPS agitator. Samples were allowed to derivatize for 4 hr at 10°C in the Gerstel MPS cool stack tray to extend the derivatization process under mild conditions for thermally labile compound prior to injection.

B. Gas Chromatography Mass Spectrometry: Samples queue was randomized. Each sample was aspirated at a fill speed of 0.5 μl/s within a solvent sandwich in the following order: 3.0 μl top air volume, 1.0 μl wash solvent plug of OmniSolv® GC hexane (EMD Millipore Corp., Darmstadt, Germany), 2.0 μl air volume above, 1.5 μl sample fill volume, 0.5 μl air volume below. Injection speed of 5.5 μl/s was set with a column headpressure of 100 kPa, total flow rate of 123 ml/min, viscosity delay of 3 s, vial penetration of 28 mm and an injection penetration of 39 mm. Samples were injected into the 30m x 0.25 mm x

0.25 μM Rtx-5® diphenyl dimethyl polysiloxane column with a fused silica 10-meter Integra-Guard® guard (Restek Corp., Bellefonte, PA) through the Gerstel cooled injection system. The started temperature for the splitless inlet was set at 150°C with an initial 0.1 min hold time and ramp rate of 1°C/s to 270°C with 20 min final temperature hold time. The Agilent 7890 GC oven was used with the following conditions: helium carrier gas, 3 ml/min inlet septum purge flow rate, 11 ml/min inlet total flow, split ratio of 10, and 280°C transfer line temperature. A temperature gradient of 2690.9 s was set for an initial 50°C with 0.20 min hold, 4°C/min ramp to 150°C with 2 min hold, 10°C/min ramp to 230°C with 0.5 min hold, and a final 27°C/min ramp to 315°C with 6 min hold. LECO Pegasus HT TOFMS was set with a mass range of 50 to 900 u, acquisition rate of 30 spectra/s, optimized detector voltage offset of 340 V, electron energy of -70 V, and the ion source temperature of 225°C.

C. Data processing: ChromaTOF GC software with True Signal Deconvolution was used to resolve spectra. The spectra were deconvoluted using a peak width of 3, segmented processing for signal-to-noise ratio above 2.0, baseline offset of 0.5 through the middle of the noise range to enhance the detection of low abundance compounds. Data points were averaged with auto smoothing. Statistical Compare software was used to align compounds across each sample and treatment groups. Normalization was performed on selected analyte for the internal standard 4-nitrobenzoic acid detected in each sample. The NIST library was used to search all masses collected based on fragmentation and retention. The database search was performed with a molecular weight range from 50 to 900, a relative abundance of base ion set at 2, minimum match similarity of 70 and area/height calculation based on the unique mass ion used to identify mass spectrum.

BrdU staining and histology

To determine hepatocyte proliferation rates, mice were injected i.p. with 100 mg/kg of 5-

bromo-2'-deoxyuridine (BrdU) (GE Healthcare) 4 hours prior to euthanize and liver paraffin sections were stained using the BrdU-in situ detection Kit (BD Biosciences, San Jose, CA). The hepatocyte proliferation index was scored as BrdU-positive nuclei per 100 cells in five nonoverlapping visual fields.

Supplemental figures

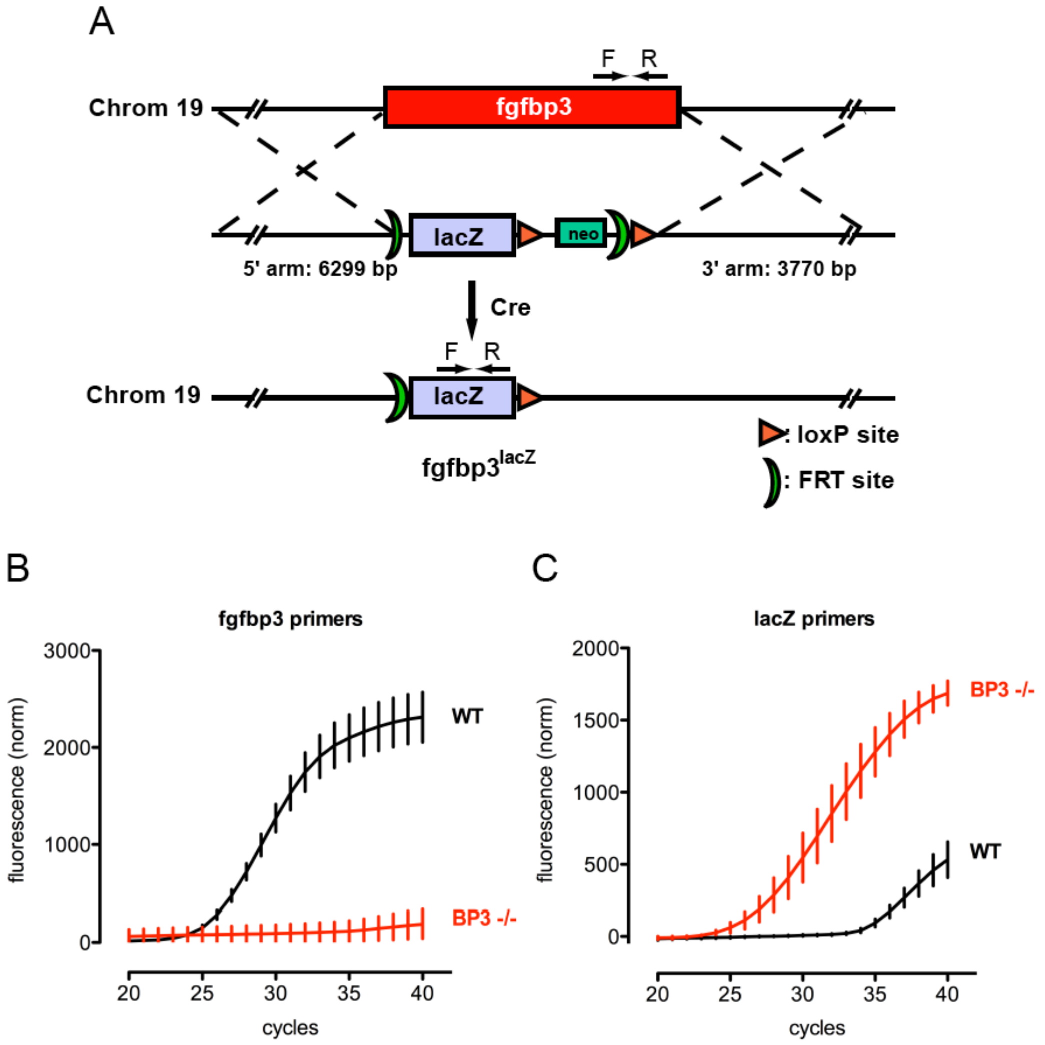

D

		p (BP3$^{-/-}$ vs WT)
**GTT**	pathologic	< 0.05
**Serum TG**	reduced	< 0.0001

Supplemental Figure 1. Generation of BP3$^{-/-}$ mice.
A. Schematic representation of the approach. The murine *fgfbp3* locus contains the complete open reading frame of the BP3 protein on a single exon that is replaced by the targeting vector containing lacZ, FRT and loxP sites. F = Forward primer; R = Reverse primer. Sequences in **Table S1**. **B,C.** Genotyping of wild type (WT) and BP3$^{-/-}$ mice by qRT-PCR using primers for mouse FGFBP3 **(B)** or inserted lacZ after Cre mediated recombination **(C)**. The fluorescence readout after the PCR cycles is shown. **(D)** Significant phenotypic alterations of BP3$^{-/-}$ mice based on a survey of the International Mouse Phenotype Consortium (IMPC) at https://www.mousephenotype.org/phenoview/?gid=125&qeid=MP:0005292.

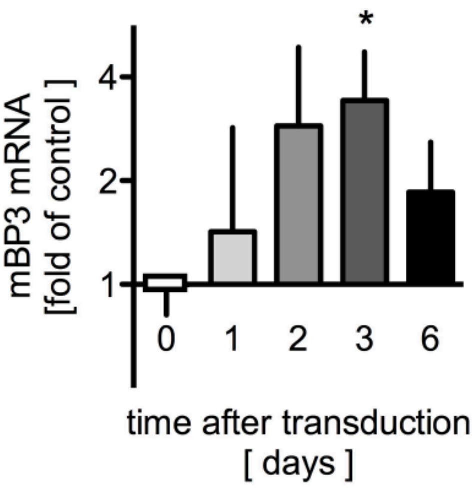

Supplemental Figure 2. Time course of expression of mBP3 after a single administration in ob/ob mice.
Change of hepatic mBP3 expression levels at 1, 2, 3, 6 days after a single dose measured by qRT-PCR. Data are expressed as fold of control (n=3).

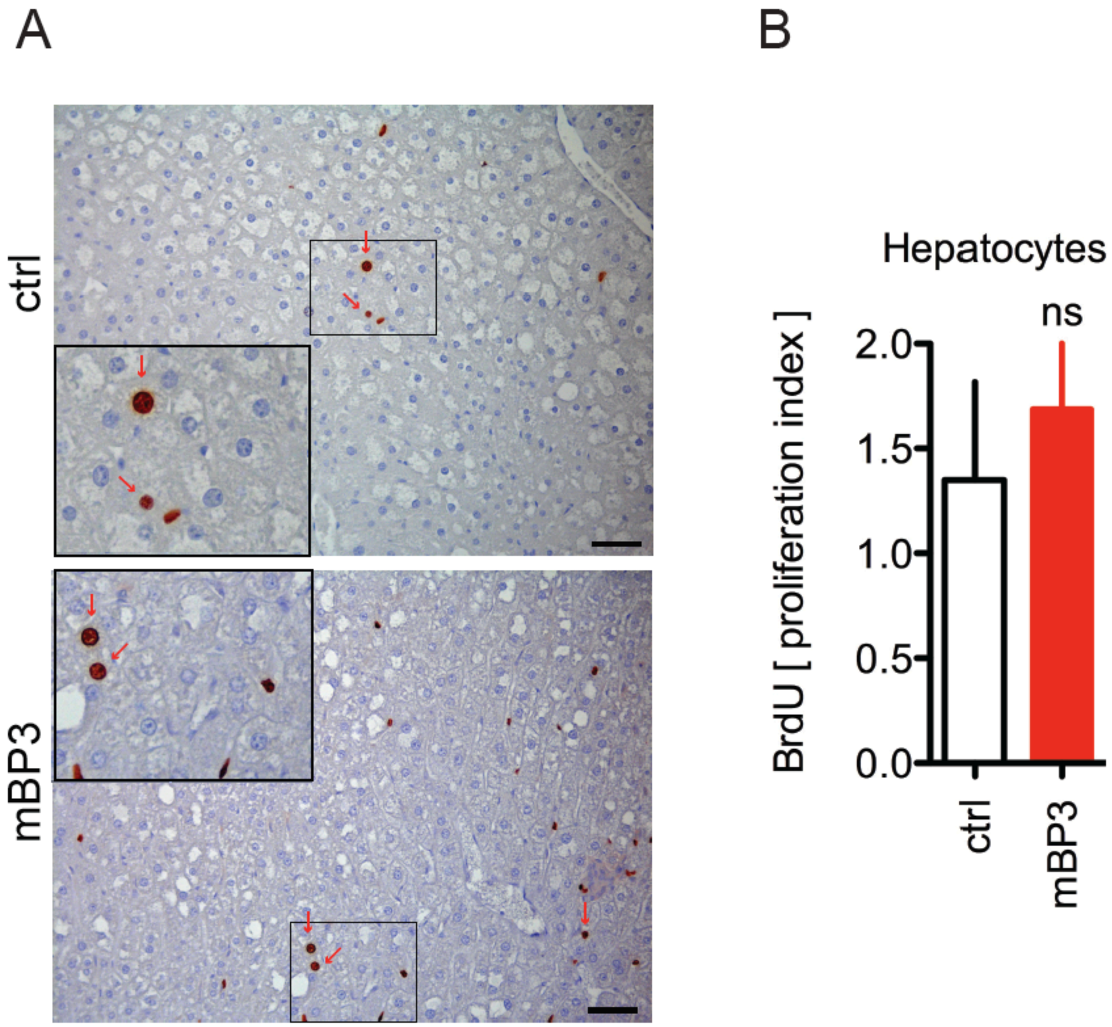

Supplemental Figure 3. Chronic mBP3 administration in ob/ob mice does not cause a mitogenic effect in liver parenchyma.

A. Representative BrdU-stained sections of livers from control and mBP3 transfected ob/ob mice. Red arrows indicate BrdU-positive hepatocytes. Scale bar: 50 μM. **B.** Proliferation index of hepatocytes (BrdU-positive fractions). All values are expressed as mean $\pm$ SEM, n = 3 (control); 6 (mBP3 transfected); ns = non significant.

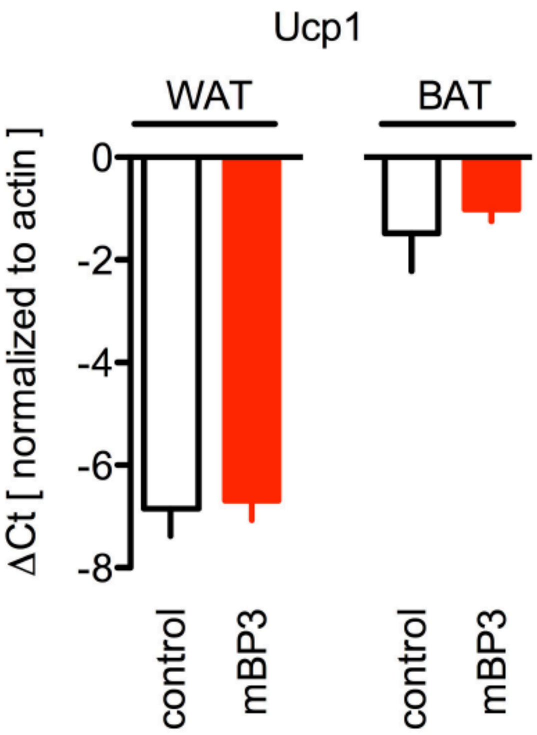

Supplemental Figure 4. Chronic mBP3 administration in ob/ob mice does not regulate Ucp1 in WAT or BAT.
Ucp1 mRNA levels in WAT and BAT of control (n=4) and mBP3 transfected (n=6) ob/ob mice. Data are normalized to endogenous beta-actin levels. Mean ± SEM, n=4 (control); 6 (mBP3 transfected).

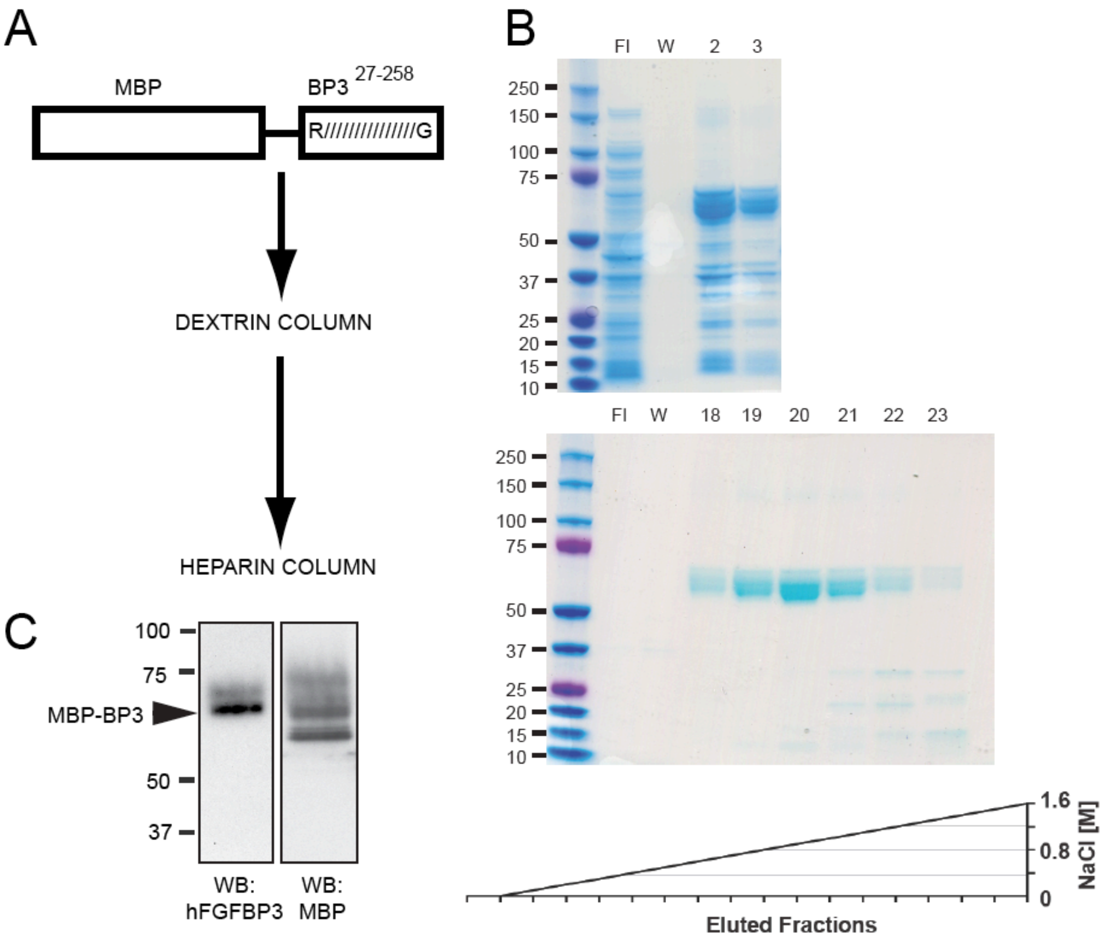

D

Secreted hBP3:
RREKGAASNVAEPVPGPTGGSSGRFLSPEQHACSWQLLLPAPEAAAGSELA
LRCQSPDGARHQCAYRGHPERCAAYAARRAHFWKQVLGGLRKKRRPCHDP
APLQARLCAGKKGHGAELRLVPRASPPARPTVAGFAGESKPRARNRGRTRE
RASGPAAGTPPPQSAPPKENPSERKTNEGKRKAALVPNEERPMGTGPDPDG
LDGNAELTETYCAEKWHSLCNFFVNFWNG

Supplemental Figure 5. Purification and detection of recombinant hBP3.
A. Schematic representation of MBP-hBP3 purification by affinity chromatography. **B.** Coomassie Blue staining of BP3 fractions purified by affinity chromatography with dextrin (upper gel) and heparin FPLC columns using a NaCl gradient, as indicated (lower gel), confirming the purity of the protein. FI = Flowthrough; W = Wash. Numbers indicate the hBP3 fraction. **C.** Western blot analysis of affinity purified MBP-hBP3 by an anti-hFGFBP3 and anti-MBP antibody. **D.** Mass spectrometry analysis of eluted hBP3.

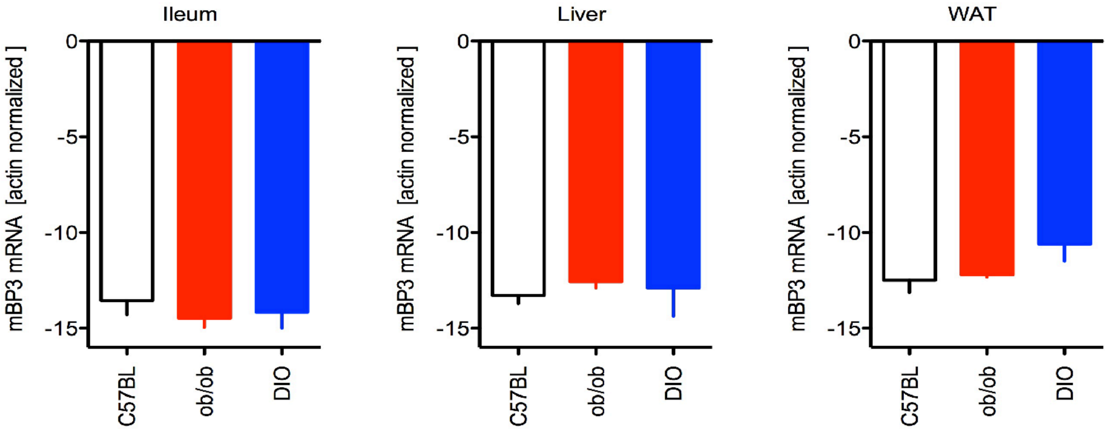

Supplemental Figure 6. BP3 mRNA expression is not different between control and obese mice.

Murine BP3 mRNA levels in ilea, livers and WAT of control, ob/ob and DIO mice (n=3-11) measured by qRT-PCR. Data are normalized to endogenous beta-actin levels. Mean ± SEM.

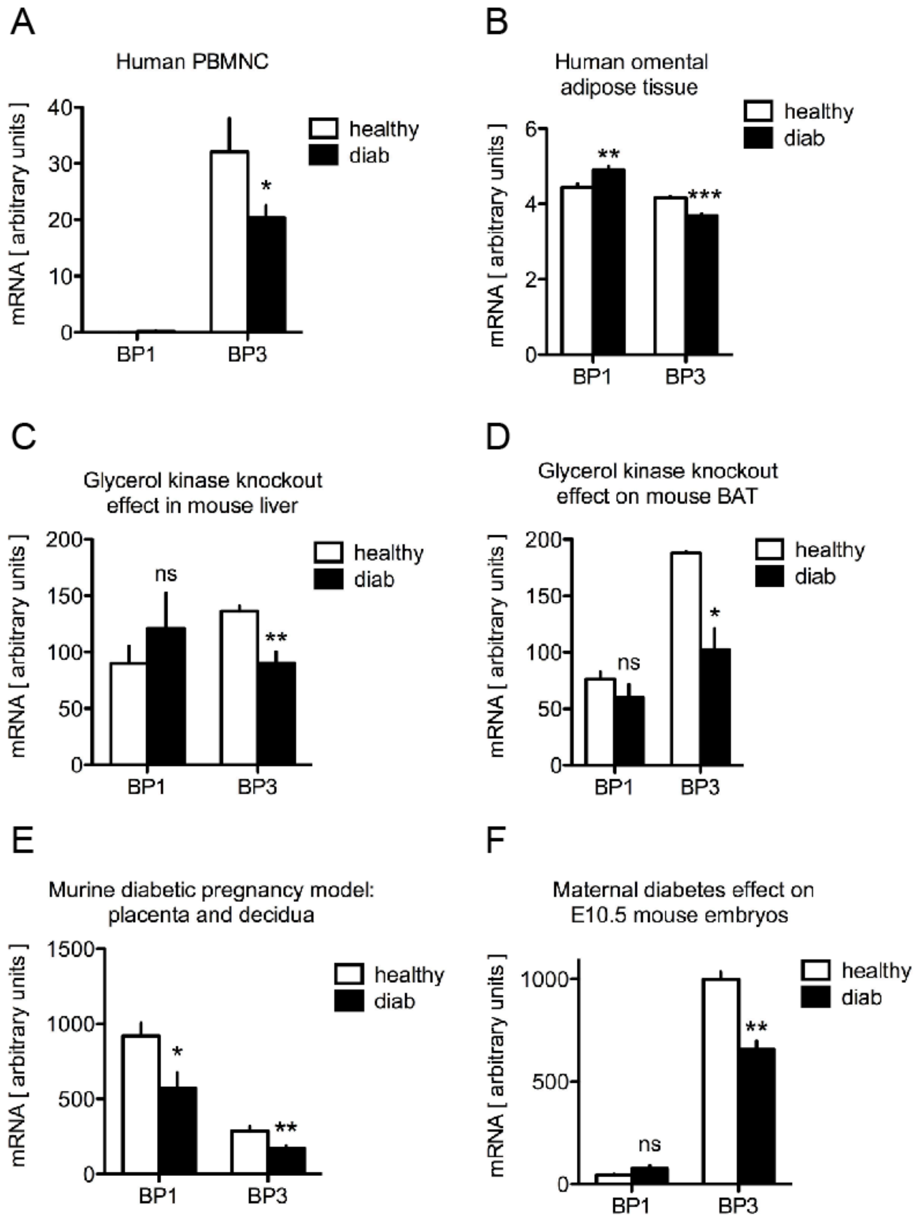

Supplemental Figure 7. BP3 mRNA expression is reduced in diabetic patients and in murine diabetic models.
FGFBP1 and FGFBP3 expression from published expression arrays of: **A.** peripheral blood mononuclear cells of healthy (n=8) and diabetic individuals (n=15) (6); **B.** omental adipose tissue of healthy and diabetic individuals (n=10) (7); **C.** liver from glycerol kinase WT (n=3) and KO mice (n=3) (8); **D.** brown adipose tissue (BAT) from glycerol kinase WT (n=3) and KO mice (n=4) (9); **E.** placentas from healthy (n=5) and diabetic (n=5) mice (10); **F.** E10 embryos from healthy (n=3) and diabetic (n=3) FVB female mothers (11). These data are from the Geo 16 Expression Omnibus (Geo) database repository (www.ncbi.nlm.nih.gov/geoprofiles/). Mean ± SEM. *, P<0.05; **, P<0.001; ***, P<0.0001.

Supplemental Table 1. qPCR primer list

	FW 5'- 3'	REV 5'- 3'
**Aacs**	GGGAGCCTGACAGCAAGAAG	CGGACAGACCAGTGGTATAAGTC
**Acac**	GATGAACCATCTCCGTTGGC	GACCCAATTATGAATCGGGAGTG
**Acas2**	AAACACGCTCAGGGAAAATCA	ACCGTAGATGTATCCCCCAGG
**Acly**	ACCCTTTCACTGGGGATCACA	GACAGGGATCAGGATTTCCTTG
**beta actin**	GGCGCTTTTGACTCAGGATTTAA	CCTCAGCCACATTTGTAGAACTTT
**Dgat1**	TCCGTCCAGGGTGGTAGTG	TGAACAAAGAATCTTGCAGACGA
**Dgat2**	GCGCTACTTCCGAGACTACTT	GGGCCTTATGCCAGGAAACT
**Fasn**	GGAGGTGGTGATAGCCGGTAT	TGGGTAATCCATAGAGCCCAG
**FGF21**	CCTCTAGGTTTCTTTGCCAACAG	AAGCTGCAGGCCTCAGGAT
**FGF15**	ACGGGCTGATTCGCTACTC	TGTAGCCTAAACAGTCCATTTCCT
**mFgfbp3**	AGCCCTTGCTAGTGAAGTCCAACT	TAGGTCTCAGTGAGCTCGGCATT
**FGFR4**	ATGACCGTCGTACACAATCTTAC	TGTCCAGTAGGGTGCTTGC
**G6pc**	CGACTCGCTATCTCCAAGTGA	GTTGAACCAGTCTCCGACCA
**Il6**	GTCACTTTGAGATCTACTCGGCAAACC	TCTGACCACAGTGAGGAATGTCCA
**KLB**	TGTTCTGCTGCGAGCTGTTAC	CCGGACTCACGTACTGTTTTT
**Lacz**	ATCTTCCTGAGGCCGATACT	CGGATTGACCGTAATGGGATAG
**Lpr1**	GTCTTCGGGGATGTGAATGTC	ACCTAAGGGTGGATCGGGTTT
**Pck1**	CTGCATAACGGTCTGGACTTC	CAGCAACTGCCCGTACTCC
**Ppargc1a**	TATGGAGTGACATAGAGTGTGCT	CCACTTCAATCCACCCAGAAAG
**Ppargc1b**	TCCTGTAAAAGCCCGGAGTAT	GCTCTGGTAGGGGCAGTGA
**Scd1**	TTCTTGCGATACACTCTGGTGC	CGGGATTGAATGTTCTTGTCGT
**Srebf1**	GCAGCCACCATCTAGCCTG	CAGCAGTGAGTCTGCCTTGAT
**Socs3**	TCTTTGTCGGAAGACTGTCAACGG	CATCATACTGATCCAGGAACTCCCGA
**Ucp1**	AGGCTTCCAGTACCATTAGGT	CTGAGTGAGGCAAAGCTGATTT

References

1. Zhang W et al. Effect of FGF-binding protein 3 on vascular permeability. *J. Biol. Chem.* 2008;283(42):28329–28337.

2. Xie B et al. Identification of the fibroblast growth factor (FGF)-interacting domain in a secreted FGF-binding protein by phage display. *J. Biol. Chem.* 2006;281(2):1137–1144.

3. Cheema AK et al. Chemopreventive Metabolites Are Correlated with a Change in Intestinal Microbiota Measured in A-T Mice and Decreased Carcinogenesis. *PLoS ONE* 2016;11(4):e0151190.

4. Kaur J. A comprehensive review on metabolic syndrome. *Cardiology Research and Practice* 2014;2014:943162.

5. Smith CA, Want EJ, O'Maille G, Abagyan R, Siuzdak G. XCMS: processing mass spectrometry data for metabolite profiling using nonlinear peak alignment, matching, and identification. *Anal. Chem.* 2006;78(3):779–787.

6. Karolina DS et al. MicroRNA 144 Impairs Insulin Signaling by Inhibiting the Expression of Insulin Receptor Substrate 1 in Type 2 Diabetes Mellitus. *PLoS ONE* 2011;6(8). doi:10.1371/journal.pone.0022839

7. Hardy OT et al. Body mass index-independent inflammation in omental adipose tissue associated with insulin resistance in morbid obesity. *Surg Obes Relat Dis* 2011;7(1):60–67.

8. MacLennan NK et al. Targeted disruption of glycerol kinase gene in mice: expression analysis in liver shows alterations in network partners related to glycerol kinase activity. *Human Molecular Genetics* 2006;15(3):405–415.

9. Rahib L, MacLennan NK, Horvath S, Liao JC, Dipple KM. Glycerol kinase deficiency alters expression of genes involved in lipid metabolism, carbohydrate metabolism, and insulin signaling. *Eur. J. Hum. Genet.* 2007;15(6):646–657.

10. Salbaum JM et al. Altered gene expression and spongiotrophoblast differentiation in placenta from a mouse model of diabetes in pregnancy. *Diabetologia* 2011;54(7):1909–1920.

11. Pavlinkova G, Salbaum JM, Kappen C. Maternal diabetes alters transcriptional programs in the developing embryo. *BMC Genomics* 2009;10:274.

Full-length blots

Figure 3C

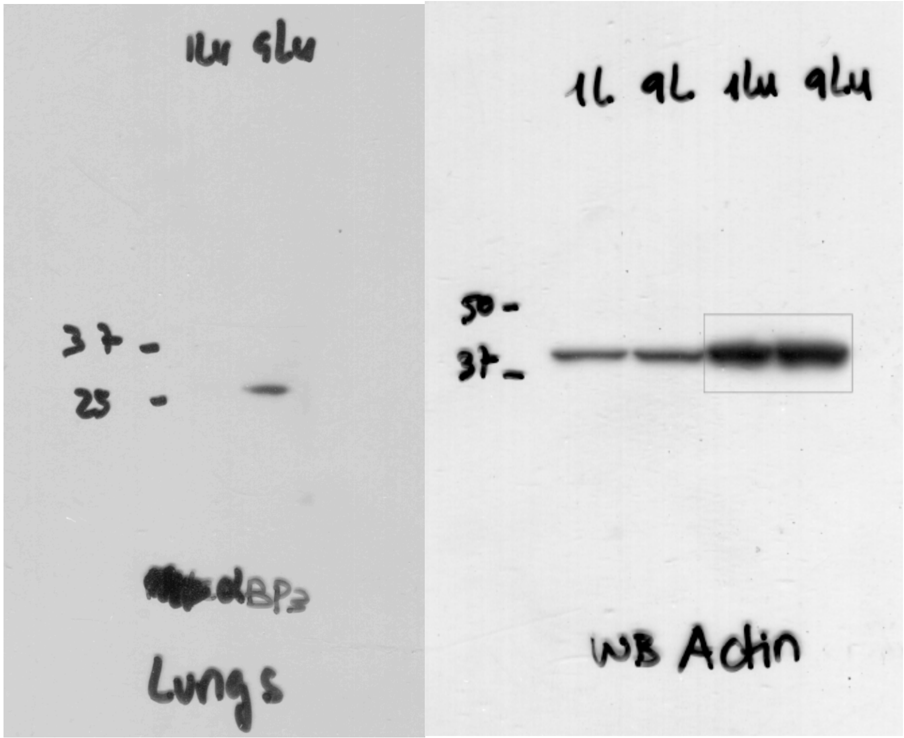

Figure 4D – left panels

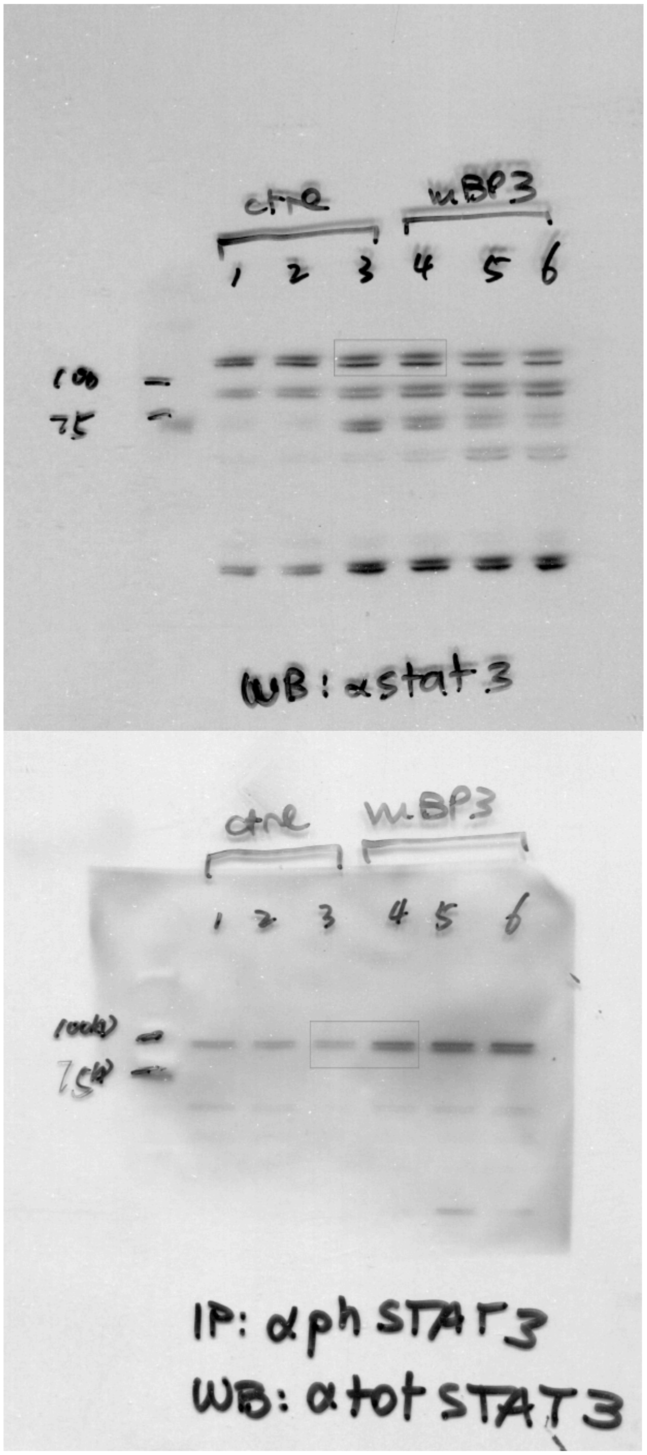

Figure 4D – right panels

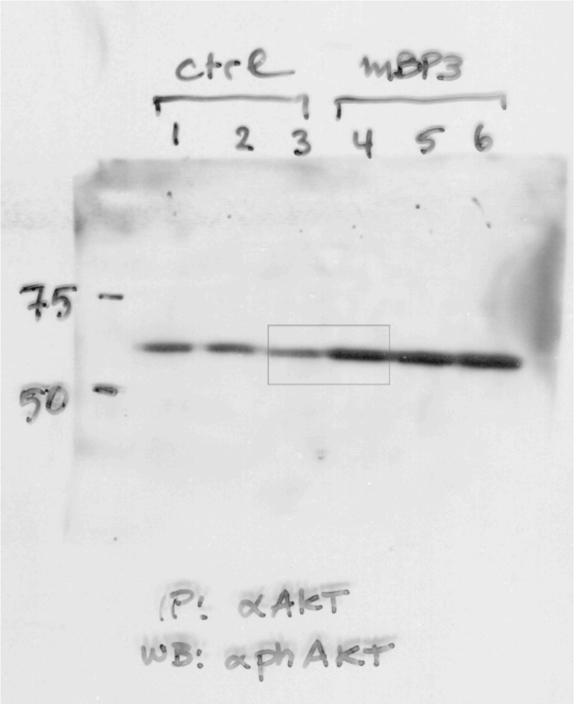

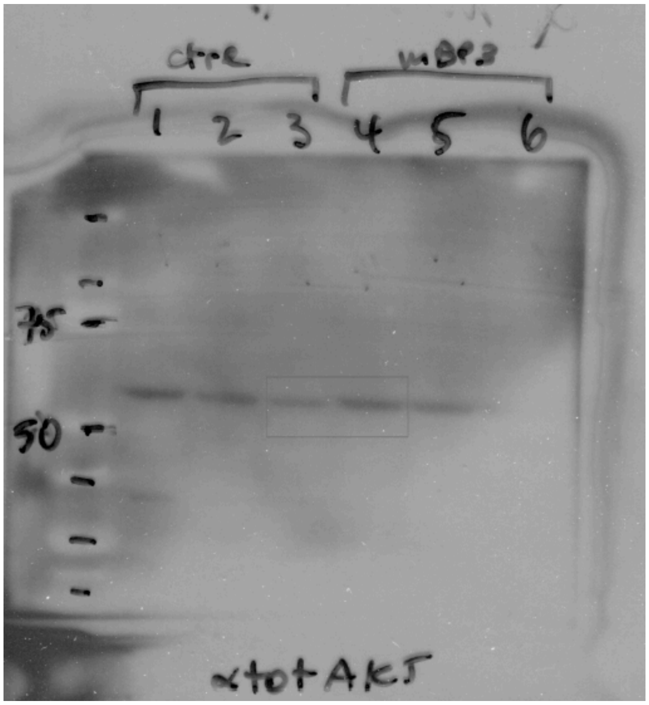

Figure 6N

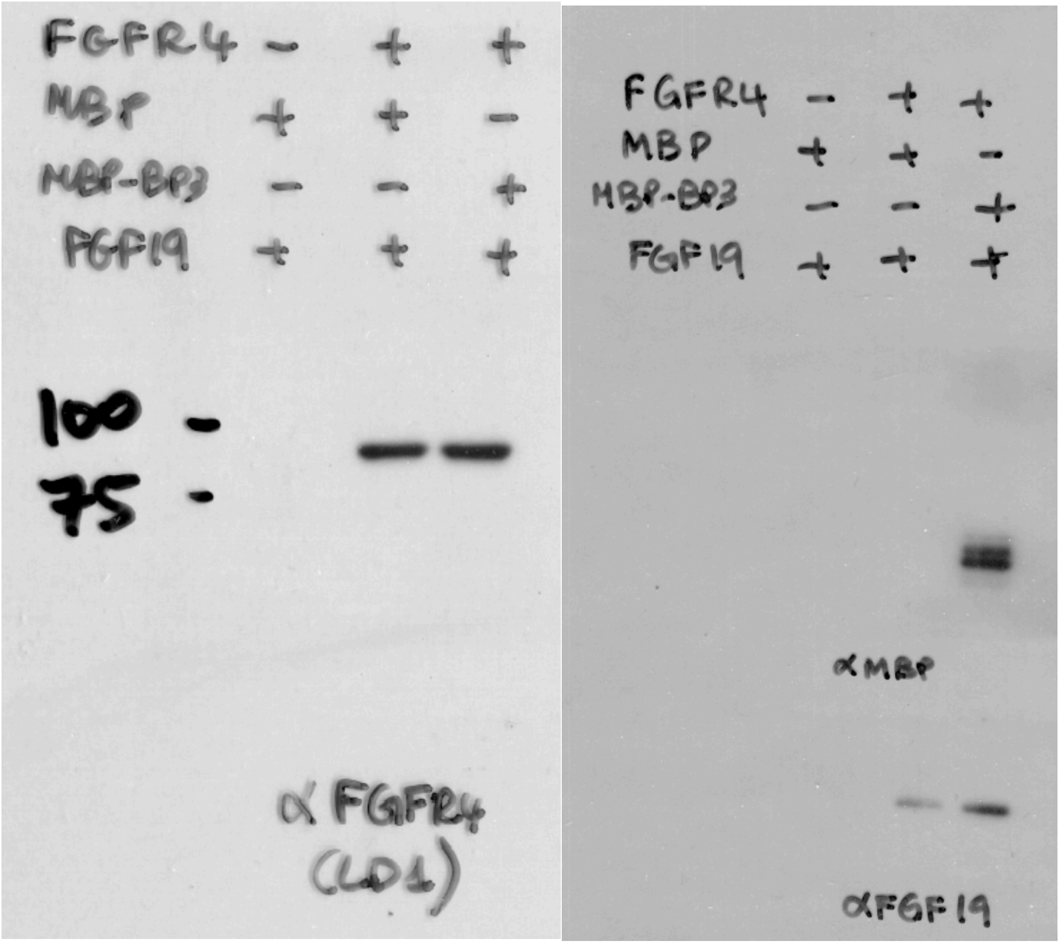
FGFR4 – + +
MBP + + –
MBP-BP3 – – +
FGF19 + + +
100
75
αFGFR4
(LD1)
FGFR4 – + +
MBP + + –
MBP-BP3 – – +
FGF19 + + +
αMBP
αFGF19

Figure 6O

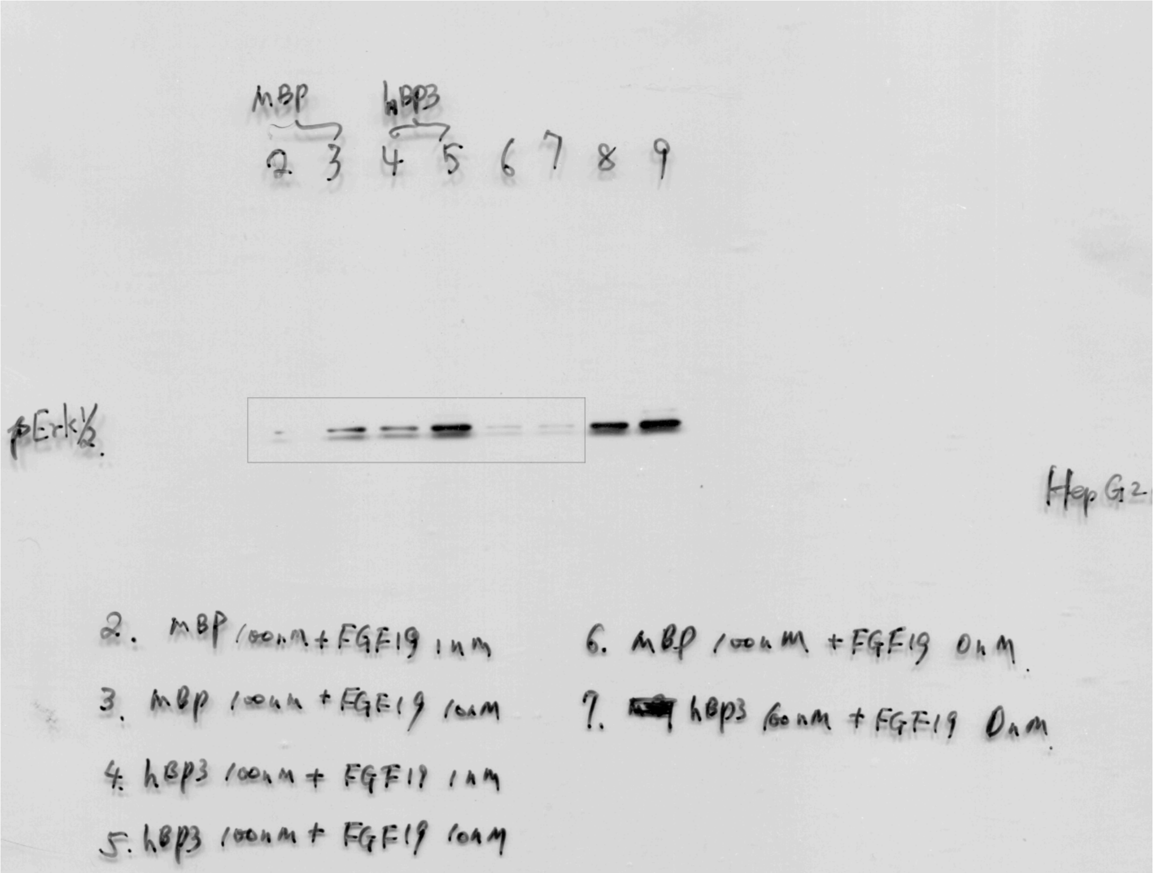
MBP
hBP3
2 3 4 5 6 7 8 9
pErk½
HepG2
2. mBP 100nM + FGF19 1nM
3. mBP 100nM + FGF19 10nM
4. hBP3 100nM + FGF19 1nM
5. hBP3 100nM + FGF19 10nM
6. MBP 100nM + FGF19 0nM
7. hBP3 100nM + FGF19 0nM

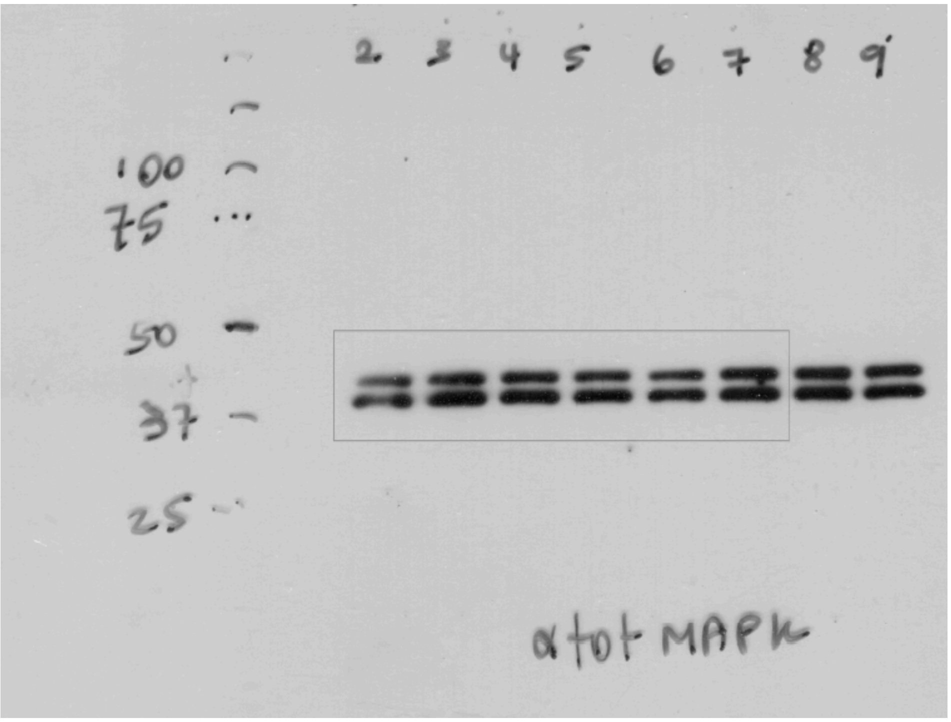
2 3 4 5 6 7 8 9
100
75
50
37
25
αtot MAPK

Figure 6P

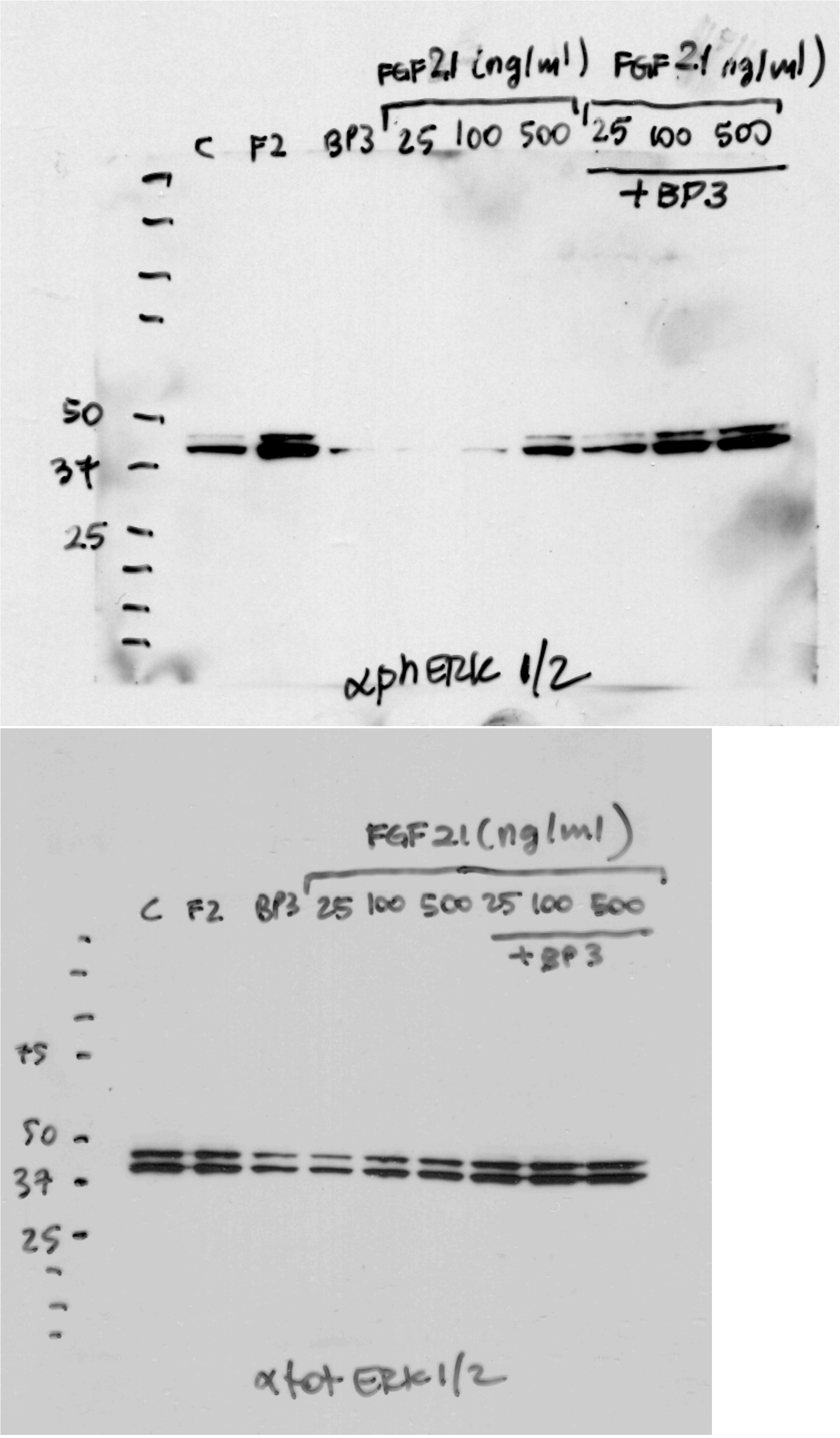
FGF21 (ng/ml) FGF21 (ng/ml)
C F2 BP3 25 100 500 25 100 500
+BP3
50
37
25
αphERK 1/2
FGF21 (ng/ml)
C F2 BP3 25 100 500 25 100 500
+BP3
75
50
37
25
αtot ERK1/2

Figure 6Q

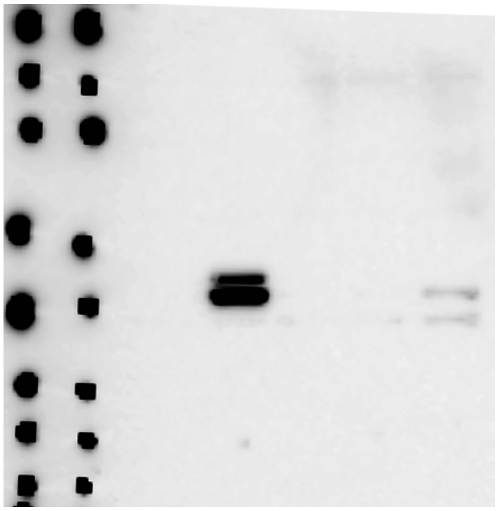

anti phospho ERK1/2

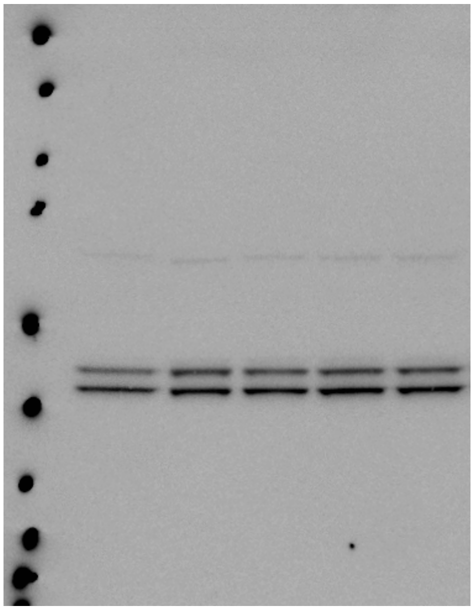

anti tot ERK1/2
